# Guide Development for eHealth Interventions Targeting People With a Low Socioeconomic Position: Participatory Design Approach

**DOI:** 10.2196/48461

**Published:** 2023-12-04

**Authors:** Jasper S Faber, Isra Al-Dhahir, Jos J Kraal, Linda D Breeman, Rita J G van den Berg-Emons, Thomas Reijnders, Sandra van Dijk, Veronica R Janssen, Roderik A Kraaijenhagen, Valentijn T Visch, Niels H Chavannes, Andrea W M Evers

**Affiliations:** 1 Department of Human-Centered Design Delft University of Technology Delft Netherlands; 2 Faculty of Social and Behavioral Sciences Leiden University Leiden Netherlands; 3 Faculty of Industrial Design Engineering Delft University of Technology Delft Netherlands; 4 Department of Rehabilitation Medicine Erasmus MC Rotterdam Netherlands; 5 Capri Cardiac Rehabilitation Rotterdam Netherlands; 6 Department of Cardiology Leiden University Medical Center Leiden Netherlands; 7 Vital10 Amsterdam Netherlands; 8 NDDO Institute for Prevention and Early Diagnostics Amsterdam Netherlands; 9 Department of Public Health and Primary Care Leiden University Medical Centre Leiden Netherlands; 10 Medical Delta Leiden University, Delft University of Technology, Erasmus University Delft Netherlands

**Keywords:** eHealth, guide, guidelines professionals, intervention development, intervention evaluation, low socioeconomic position, low socioeconomic status, risk groups, tailored care

## Abstract

**Background:**

People with a low socioeconomic position (SEP) are less likely to benefit from eHealth interventions, exacerbating social health inequalities. Professionals developing eHealth interventions for this group face numerous challenges. A comprehensive guide to support these professionals in their work could mitigate these inequalities.

**Objective:**

We aimed to develop a web-based guide to support professionals in the development, adaptation, evaluation, and implementation of eHealth interventions for people with a low SEP.

**Methods:**

This study consisted of 2 phases. The first phase involved a secondary analysis of 2 previous qualitative and quantitative studies. In this phase, we synthesized insights from the previous studies to develop the guide’s content and information structure. In the second phase, we used a participatory design process. This process included iterative development and evaluation of the guide’s design with 11 professionals who had experience with both eHealth and the target group. We used test versions (prototypes) and think-aloud testing combined with semistructured interviews and a questionnaire to identify design requirements and develop and adapt the guide accordingly.

**Results:**

The secondary analysis resulted in a framework of recommendations for developing the guide, which was categorized under 5 themes: development, reach, adherence, evaluation, and implementation. The participatory design process resulted in 16 requirements on system, content, and service aspects for the design of the guide. For the system category, the guide was required to have an open navigation strategy leading to more specific information and short pages with visual elements. Content requirements included providing comprehensible information, scientific evidence, a user perspective, information on practical applications, and a personal and informal tone of voice. Service requirements involved improving suitability for different professionals, ensuring long-term viability, and a focus on implementation. Based on these requirements, we developed the final version of “the inclusive eHealth guide.”

**Conclusions:**

The inclusive eHealth guide provides a practical, user-centric tool for professionals aiming to develop, adapt, evaluate, and implement eHealth interventions for people with a low SEP, with the aim of reducing health disparities in this population. Future research should investigate its suitability for different end-user goals, its external validity, its applicability in specific contexts, and its real-world impact on social health inequality.

## Introduction

Global progress in improving health has been challenging. For example, the burden of noncommunicable chronic diseases, such as cardiovascular disease, diabetes, and obesity, is higher among racial, ethnic, and lower socioeconomic (below-average occupational position, educational level, and income) groups [1-5]. A low socioeconomic position (SEP) is associated with a higher prevalence of unhealthy lifestyles compared to a high SEP [6-8]. A large segment of our society comprises people with a low SEP. For instance, in the Netherlands in 2019, there were 574,000 households with incomes below the low-income threshold, accounting for 7.7% of all households [9]. Studies suggest that people with a low SEP face many challenges that impact their health [8,10]. For example, people with a low SEP may have low literacy or live in poverty under stressful conditions such as money-related stress, unfavorable work environments, or unemployment [11]. Various efforts have been made to reduce the incidence of noncommunicable chronic diseases in current societies through lifestyle change, including the use of eHealth interventions. eHealth interventions, such as monitoring devices, web-based communication platforms, and persuasive applications, have proven effective in changing behavior and promoting a healthy lifestyle when tailored appropriately toward the needs and preferences of the individual [12,13].

The use of smartphones and social media is not exclusive to people with a high SEP. These technologies have gained acceptance among people with a low SEP, particularly among less educated working young adults [14]. Recognition of the benefits of eHealth for lower-SEP groups is growing [13,15-17]. Many studies acknowledge that tailoring eHealth interventions to specific needs improves patient engagement and leads to more durable behavior changes [12,13]. People with a low SEP can particularly benefit from the asynchronous communication and multimedia patient education provided by eHealth [18], as they report lower satisfaction with patient-provider communication than those with a higher SEP [19]. eHealth also has the potential to improve access to care [20] by reducing barriers such as the need for long-distance travel and its costs and allowing for personalized consideration of social, economic, and physical factors that may impact their lifestyle [21]. Finally, information individualized toward a person’s level of health literacy can improve knowledge and be more readily recalled [22]. Despite the potential benefits of eHealth for people with a low SEP, there is a significant lack of clarity in this area [23]. The available information on the effective components of eHealth interventions for such groups is limited, leaving room for doubt and uncertainty. For example, a scoping review highlighted variations in the components of eHealth interventions and the barriers and facilitators involved in their development and implementation [23].

Sufficient practical guidance that can be directly applied by professionals (eg, eHealth developers, researchers, health care providers, and policy makers) in the field is missing. What does exist are some basic approaches to making eHealth more accessible to people with a low SEP. These include adapting the content of the interventions by avoiding medical terminology, using more pictures, and using simple user interfaces [24]. However, while improving the readability of and accessibility to information is important, achieving successful behavior change requires tailoring interventions that extend beyond focusing on simplicity and understandability and improve the deeper factors related to motivation on social, cultural, and economic levels [25].

Moreover, there are challenges in designing interventions for this target group. First, professionals often see eHealth as a one-size-fits-all solution, but this approach can exclude lower-SEP groups [26]. While there is knowledge available about involving these groups, for example, through participatory methods [17,27,28], they are often not implemented due to the limited availability of resources, expertise, knowledge, and awareness about lower-SEP groups within a project or team [29].

Second, although there is abundant knowledge on barriers to and facilitators for including the target group in interventions, there are still difficulties faced by professionals in the field, including eHealth developers, researchers, and health professionals, in reaching people with a low SEP and ensuring their adherence to eHealth interventions. Interventions that are not tailored toward the needs, skills, and preferences of the target group can and will be less effective [5,22,30-33]. To enhance the development and adaptation of eHealth interventions for people with a low SEP, it is essential to acknowledge the current challenges faced by professionals in using informational resources and tools. These difficulties include information overload and comprehension difficulties [34,35], difficulties in aligning theory with practice [36-39], and the lack of a human-centered approach leading to generalized information [38]. The World Health Organization provides guidelines for digital interventions aimed at enhancing health systems [40]. However, despite guidelines being comprehensive and credible, professionals often struggle with the practical implementation of these guidelines. The guidelines by the United Nations [41] provide more applicable guidance, yet they focus mainly on skills and literacy barriers faced by end users without assisting professionals during the development process. To the best of our knowledge, there is currently no accessible and applicable guidance available to assist professionals throughout the process of developing eHealth interventions for people with a low SEP.

The objective of this study was to address the challenges faced by professionals in developing, adapting, evaluating, and implementing eHealth interventions (eg, lifestyle interventions) for people with a low SEP. To overcome these challenges, our aim was to develop a comprehensive guide that supports professionals throughout this process. This guide is intended to provide guidance and assistance to professionals working in the field of eHealth (eg, lifestyle interventions) across a wide range of settings, such as health care facilities (eg, hospitals and cardiac rehabilitation) and individual self-management for chronic disease. We aimed to ensure that the guide is user-friendly and accessible by identifying and incorporating design requirements derived from the needs and preferences of professionals in relation to such a guide.

## Methods

### Study Design

**Figure 1:**
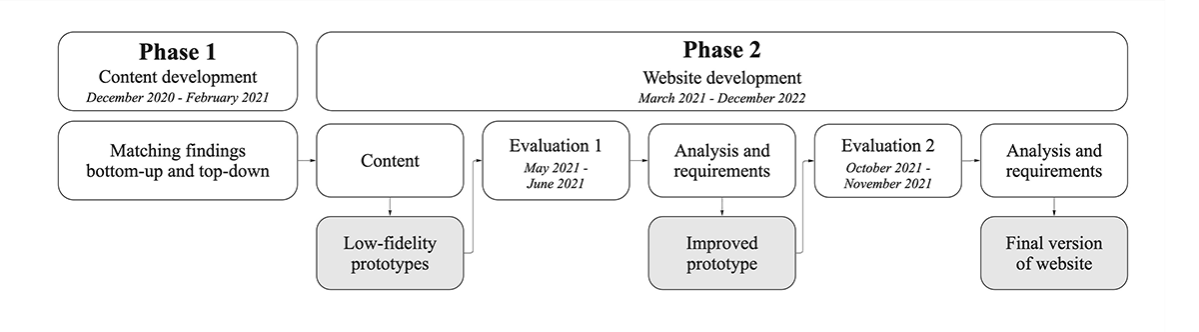
Schematic overview of phases, methods, and iterations.

This study uses a 2-phase qualitative research approach that includes a secondary analysis of existing data and a participatory design process (Figure 1). In the first phase, we performed a secondary analysis of data from 2 existing qualitative and quantitative studies. In the second phase, we adopted a participatory design process, involving the prospective end users (professionals who would be using the guide) directly in the development process.

### Procedure and Materials

#### Phase 1: Secondary Analysis and Development of the Content

The goal of this phase was to develop the content and information structure of the guide. Activities included secondary analysis with the goal of combining data from previously conducted Delphi and community-based participatory research (CBPR) studies. The Delphi study was performed with professionals and identified their experienced barriers and facilitators regarding eHealth development, reach, adherence, implementation, and evaluation for people with a low SEP (top-down) [29]. The CBPR study was conducted with people with a low SEP and resulted in different profiles of their attitudes toward health, health care, and eHealth (bottom-up) [42]. Analyzing these studies through a qualitative secondary analysis allowed us to extract, combine, and synthesize insights that we used to develop the content and structure of the guide [43]. The combination of bottom-up and top-down approaches represents an innovative methodology that is not widely used. eHealth interventions exhibit limited alignment with the needs and preferences of people with a low SEP, thereby resulting in their underuse by this target group [16,31]. Professionals are crucial in adjusting these interventions to meet the target population’s needs. By integrating the perspectives of professionals (as guide users) and people with a low SEP who engage with eHealth interventions, we can develop a comprehensive guide that substantially enhances interventions for this group.

**Figure 2:**
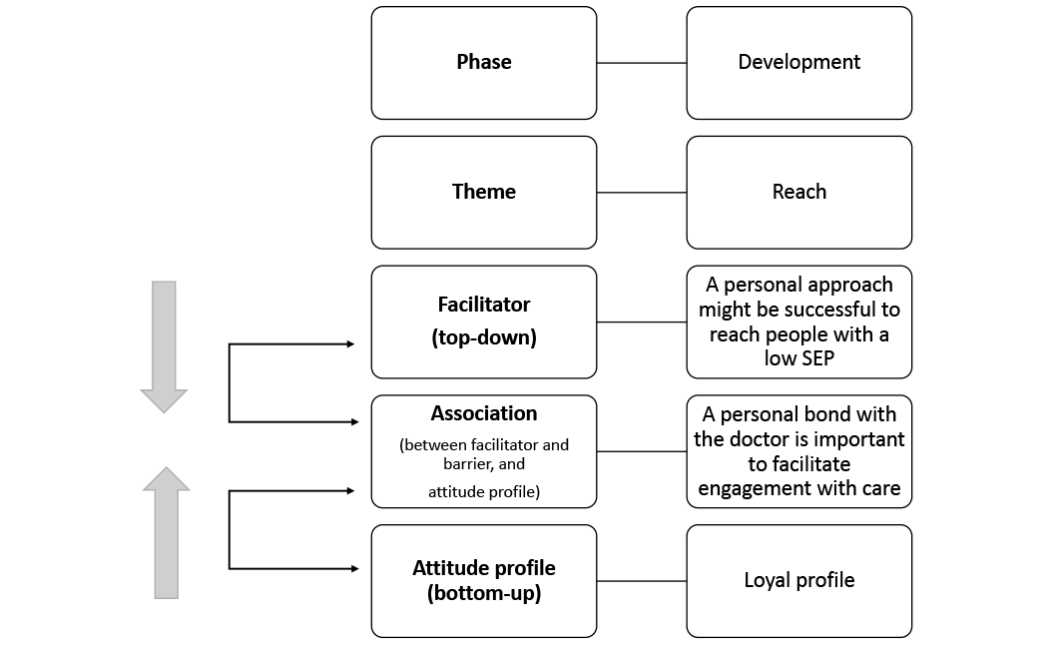
An example of an association made between a facilitator identified through the top-down approach and an attitude profile identified through the bottom-up approach. SEP: socioeconomic position.

The secondary analysis embodied the analysis, discussion, and synthesis of data obtained from the previous studies. For the analysis, both first authors (JSF and IAD) independently analyzed the barriers and facilitators of the Delphi study [29] and considered which profiles of the CBPR study [42] could be influenced by them. For example, the Delphi study identified a barrier where professionals lacked sufficient knowledge about the daily lives of people with a low SEP, which aligned with attitude profiles from the CBPR study characterized by difficulty in comprehending written materials and limited digital skills. Another example is provided in Figure 2. Both authors independently documented their associations in Microsoft Excel.

For the discussion, the first authors discussed their associations and documented their alignment using color coding in Excel, making distinctions between “full alignment,” where both authors found the same association; “to be discussed,” where alignment did not match; and “not applicable,” where both authors did not find an association between the 2 studies. A second discussion round followed, in which both authors discussed the “to be discussed” associations and developed a mutual agreement on the corresponding association. Finally, during synthesis, the first authors developed the association scheme (Multimedia Appendix 1).

#### Phase 2: Participatory Development of the Guide

The goal of the second phase was to use the findings from phase 1 to create a guide through an iterative process with end users. In this phase, we used a participatory design approach. Participatory design, also known as co-design, is an approach that emphasizes the active involvement of end users in a design or development process to ensure that the result meets their needs [44,45]. Participatory design is often used in an iterative manner. The iterative process allows for continuous reflection on intermediate results and enables ongoing learning to make improvements. For this reason, we engaged the professionals in 2 rounds of prototype evaluation (simple representation of the final product) of the guide.

We aimed to recruit end users that would eventually make use of the guide: professionals working within the development, adaptation, implementation, or evaluation of eHealth. Consequently, we did not involve people with a low SEP to evaluate the guide, primarily because they are not the direct users of the guide. The inclusion criteria for participation were that the professional should have experience with eHealth within their role as well as experience with developing, adapting, evaluating, and implementing eHealth in the context of low-SEP populations. We used scientific literature and input from the Delphi study to identify the roles of professionals to be included: policy officers, health care providers, eHealth developers, and researchers. To contact these professionals, we used expert recommendations and snowball sampling. For the first evaluation session, we invited at least 1 of each type of professional through email. For the second evaluation session, we invited, through email, professionals from round 1 as well as new professionals. In web and usability design, the rule of thumb is that testing with just 5 participants can uncover at least 80% of user insights when the aim is to generate insights rather than validate them [46]. Although this number can vary depending on the project [47], we followed this guideline by targeting a minimum of 2 participants for each role to ensure a well-rounded set of user insights. It is important to note that qualitative research aims to understand the human experience in a comprehensive, nuanced manner. While it may not quantify the prevalence of a specific experience or need in the same way as quantitative research, it aims to explore the depth, meaning, and significance of such experiences or needs within a specific context or population.

#### Development of Prototypes

For the first prototype evaluation session, we developed 3 low-fidelity (quick and dirty) prototypes of the website. These prototypes provoked our participants to comment on the ideas instead of specific features (eg, colors used and button placement). To develop these prototypes, we gathered inspiration on navigation, credibility, tone of voice, applicability, communication style, and user perspective from existing tools (eg, guidelines and roadmaps) on eHealth development, inclusivity, low SEP, low health literacy, accessibility design, and general design using the Miro whiteboard platform [48]. We identified reoccurring elements, such as do’s and don’t’s, personas, examples, and tips. As a final step, we synthesized the individual elements into 3 clickable prototypes in Microsoft PowerPoint (Multimedia Appendix 2).

For the second prototype evaluation session, we developed an improved prototype based on the results of the first evaluation using the Wix website builder [49] (Multimedia Appendix 2).

#### Evaluation of the Prototypes and Content

Both evaluation rounds comprised individually conducted semistructured interviews and used the think-aloud method, where participants verbalized their thoughts, to gather information. Semistructured interviews are an effective approach for collecting information, while the think-aloud method serves as a valuable technique to gain insights into user thoughts and perceptions [50]. These methods enabled us to understand the target group better and contributed to the creation of an appealing prototype [51]. The first evaluation was performed to determine professionals’ goals and needs based on content, system, and service level. The second evaluation was conducted to determine how the participants valued the recommendations (content) and to get an indication of user acceptance of the prototype.

We ran a pilot for both evaluation rounds with 2 researchers to refine the protocol. The first evaluation was conducted on the internet (in accordance with COVID-19 regulations), while the second evaluation was conducted either on the internet (Microsoft Teams; Microsoft Inc) or face-to-face based on the preference of the participant. The sessions lasted between 45 and 60 minutes and were recorded using a voice recorder or through Microsoft Teams. The determination of the number of interview sessions conducted in each evaluation round was based on the input received from the participants in the study, which played a crucial role in guiding this decision. After consultation with the research team, it was concluded that both evaluation sessions yielded sufficient data to proceed with the development of the website. In the first evaluation round, we started asking participants about their background information, including their role, age, experience with eHealth, and the target group. Subsequently, we discussed the 3 low-fidelity prototypes. We first introduced the participant to a predetermined scenario. The scenarios were written according to different roles: eHealth developer, researcher, and health care provider (Multimedia Appendix 3). An example scenario for researchers was:

Imagine you are involved in a study on eHealth and people with a low SEP. The problem is there is too much information available. You are looking for a central place to find all the information. A colleague tells you about an online guide for the development of eHealth interventions for people with a low SEP. You decide to visit. Your goal is to quickly get a good overview of the information and to quickly access the information source through the website.

We also asked participants to try each prototype and offer a brief verbal evaluation. In the last part, we asked questions about the prototypes and the content: “Which prototype do you like the most? Which specific themes or topics do you want to see in the guide?” The interview guide is included in Multimedia Appendix 3.

In the second evaluation round, we again started with collecting relevant background information from the new participants. Thereafter, we asked all participants to execute 5 tasks while verbalizing their thoughts: (1) explore the pages, (2) find a barrier on a specific topic, (3) find an associated facilitator, (4) find the associated practical tips, and (5) find the associated user perspective. Finally, at the end of the interview, we administered a short questionnaire as an assessment tool to evaluate the prototype and assess the likelihood of acceptance of the final guide among study participants. We developed this questionnaire based on the usability, satisfaction, and ease-of-use (USE) questionnaire [52]; the unified theory of acceptance and use of technology (UTAUT) questionnaire [53]; and the internet evaluation and utility questionnaire (IEUQ) [54,55] questionnaires. It comprised 13 questions regarding the intention to use, usefulness, and usability of the design, as well as the relevance, understandability, and trustworthiness of the content (Multimedia Appendix 3). The items were rated on a 5-point Likert scale, ranging from 1 “certainly not” to 5 “certainly” [56]. To analyze the questionnaire responses, we calculated the percentages (means and SDs) and classified scores as negative (1 or 2), neutral (3), or positive (4 or 5) for each item [57,58].

### Data Analyses

Since this study adopted a participatory approach, we used the data from the first session to develop the prototype and the data from the second session to refine the prototype guide [59-61]. Thematic analysis was applied to both sets of data, following the method outlined by Braun and Clarke [62]. The first authors coded and themed data separately using the qualitative data analysis Atlas.ti software (version 9; ATLAS.ti Scientific Software Development GmbH) [63]. Themes were coded through open coding and thereafter categorized through axial coding within 3 predetermined categories: service, system, and content, as provided in Kelders et al [64]. The system category describes the website’s layout and information structure. The content category describes the usefulness of the information and the understandability of the text on the website. The service category describes the process of care given by the website, including credibility and long-term implementation.

We identified recurring themes and items of interest that offered insights into the wishes and needs of professionals. Initial codes and themes were discussed in several sessions, and the results were then compared and merged by consensus. The codes were also given a positive, neutral, negative, and recommendation label. After each interview round, we used the themes resulting from the analysis to synthesize a list of requirements for the next prototype. For this, we examined the frequency of occurrence and the number of participants who mentioned the themes. Positive themes related to aspects that should be kept and elaborated upon. Negative aspects were paired with recommendations for improvement. The requirements were related to content, system, and service and encompassed the most important needs, wishes, and preferences of the participants.

Background information was analyzed using descriptive summary statistics. Quantitative data about the acceptance of the prototype in the second evaluation session were descriptively analyzed using SPSS Statistics 25 (IBM Corp) [65].

### Ethical Considerations

Ethical approval for the study was obtained from the Human Research Ethics Committee of the Delft University of Technology (approval 1495). All participants were informed about the study and signed an informed consent form before participating. Participants were reimbursed for their participation with a €15 (US $15.88) donation to a low-SEP–oriented charity.

## Results

### Phase 1: Content and Information Structure

**Figure 3:**
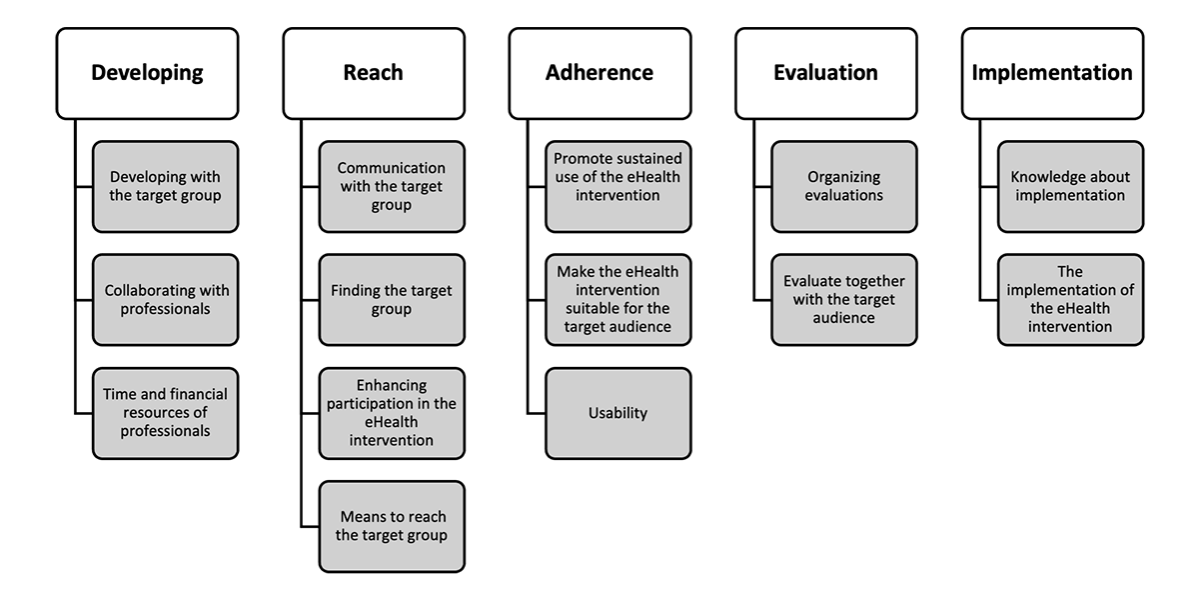
Overall information structure of the content of the guide resulting from the secondary analysis.

Based on the secondary analysis, an association scheme was created to present the content and information structure categorized under 5 different aspects of eHealth development (development, reach, adherence, evaluation, and implementation). Within each category (eg, development), different themes could be found that relate to this category. Within each theme, associations could be found between barriers, facilitators, and attitude profiles. For instance, under the development aspect, themes such as “developing with the target group” can be identified (Figure 2). The overall information structure is visually presented in Figure 3.

### Phase 2: Design of the Inclusive eHealth Guide

The results from the participatory process with potential end users (professionals) of the guide consist of several recommendations on system, content, and service aspects of the guide. These insights were subsequently translated into an interactive web-based guide aimed at facilitating eHealth development.

### Participants

In total, 11 professionals participated in this study. In the first interview session, 7 professionals participated. A total of 10 participants contributed to the second interview session; 6 of them also participated in the first interview session. The participants had experience working with people with a low SEP and consisted of eHealth developers, health care providers, researchers, and policy officers (Table 1).

**Table 1:** Participant characteristics.

Participant	Interview session	Sex	Role	eHealth experience (years)	Activities
1	1	Male	Developer	4	eHealth intervention development
2	1 and 2	Female	Health care provider and researcher	8	Scientific research, eHealth intervention development, and health care practice
3	1 and 2	Female	Researcher	6	Scientific research
4	1 and 2	Female	Policy officer	3	Policy development
5	1 and 2	Male	Developer	10	eHealth intervention development
6	1 and 2	Male	Developer	10	eHealth intervention development
7	1 and 2	Female	Policy officer and developer	9	eHealth intervention development, eHealth intervention application, and policy development
8	2	Female	Researcher	5	Scientific research and eHealth intervention development
9	2	Male	Developer	19	eHealth intervention development
10	2	Male	Health care provider	7	eHealth intervention application and health care practice
11	2	Female	Policy officer and developer	Not available	Policy development and eHealth intervention development

### Requirements Based on Prototype Evaluation

The analysis of both interview rounds generated 96 themes, with 39 themes arising from interview 1 and 57 themes from interview 2. A detailed overview of the themes extracted from interviews 1 and 2 is provided in Multimedia Appendix 4. The subsequent synthesis resulted in the formulation of 16 requirements for the final guide design, covering content, system, and service aspects. Table 2 presents examples of participant quotes and the resulting requirements.

Regarding the system category, the guide is required to have (1) an open navigation strategy that allows different types of users to reach their desired information through multiple pathways instead of a predetermined (closed) navigation route. This should be facilitated by a (2) starting scheme that serves as both a starting and come-back “reference” point to improve the navigation experience. From this starting point, the user should be directed deeper into more detailed and (3) specific information about, for example, the barriers and facilitators. (4) Visual elements should be included, while the amount of text should be reduced, and the overall system should be made visually appealing to make the navigation more enjoyable. Both barriers and facilitators needed to follow (5) a concurrent presentation rather than a sequential presentation that highlighted barriers before facilitators. A balanced depiction is needed to avoid any dominance of one over the other. Long pages should be subdivided and categorized into more digestible (6) shorter separate pages.

For the content, the guide is required to provide (7) comprehensible information that is supplemented and made credible with (8) scientific evidence, for example, by referring to literature. The content should provide (9) a realistic user representation to improve empathy toward the target group. This user information should be short and be accompanied with interpretable (10) abstract user information (eg, tell exactly what the users’ barriers are instead of “hiding” them in a story). The barriers and facilitators should be accompanied by information about the (11) practical application, for instance, by providing examples and practical tips. Finally, (12) the tone of voice should be personal and informal to improve the persuasiveness of and engagement with the content.

Service requirements included improving (13) the suitability for different professionals, such as health care providers and developers, which have different needs and goals. In addition, a key requirement was to ensure (14) the long-term viability of the website. This involves considering the costs associated with maintaining the website and ensuring that the information present is constantly up to date. To achieve this, the guide should become (15) a dynamic community hub that connects various instances, people, and research groups for sharing knowledge. Finally, there is a need for increased (16) focus on the implementation of intervention development. This was deemed essential for ensuring the success and impact of interventions within the target group.

**Table 2:** Content, system, and service requirements based on the first and second interviews.

Requirement			Description		Quote
**System**					
	1. Open navigation	Present an open overview about the information structure and possible navigation strategies.		“In terms of user experience, I would like to navigate through different pathways. That would be my most important requirement.” [P5]	
	2. Starting scheme	Provide both a starting point and a persistent reference point for maintaining an overview while navigating		“I saw that overview at the beginning; I actually keep going back to it.” [P11]	
	3. Specific information	Provide direction down a specific path, allowing the user to switch quickly between generic and specific information		“It feels like a nice step, you have 2 new categories within a category. So, you are talking about reach, and you can see for yourself which aspect of reach is important to me.” [P4]	
	4. Visual elements	Incorporate visual elements to enhance the overall appeal and user experience.		“If you have different icons, this would be useful.” [P9]	
	5. Concurrent presentation	Present barriers and facilitators concurrently instead of sequentially to avoid dominance of one over the other.		“...and indeed, what I just said, seeing a lot of barriers among each other is a deterrent, all those exclamation marks among each other.” [P6]	
	6. Shorter, separate pages	Make use of more categories and shorter pages to improve the reader’s retention		“You really don’t have to go down that much. I don’t like scrolling that much.” [P10]	
**Content**					
	7. Comprehensible information	Information should be clear and understandable, avoiding technical jargon		“For the attitude part it was not directly clear for me that you were talking about the patient, till I started reading.” [P1]	
	8. Scientific evidence	Information should be backed up by scientific evidence to improve its credibility		“That I can trace back: Where does it come from? Where is it based on?” [P6]	
	9. Realistic user representation	Use realistic user representations to enhance empathy instead of relying on fictional quotes and examples		“I would like to see real practical examples in case studies.” [P5]	
	10. Abstract user information	Accompany user-related information with more abstract statements that are easier to interpret		“My mind works better with more abstract or conceptual information than with examples.” [P4]	
	11. Practical application	Include practical guidance on how to implement the recommendations through concrete examples and practical tips		“I would have expected more guidance, say I visit the website and want to adapt something, how should I do it?” [P2]	
	12. Informal tone of voice	Use a personal and informal tone of voice to improve the persuasiveness of the content		“It is very much written in policy language.” [P7]	
**Service**					
	13. Suitability for different professionals	Ensuring accessibility and usefulness for different groups of professionals (eg, developers and health care providers)		“Well, I do think that it would appeal to health care practitioners towards people they see in their daily practice.” [P7]	
	14. Long term viability	Keeping the website up to date, maintained, and disseminated		“This is one of the most challenging aspects; you can make a beautiful website, but who is going to visit it? Who knows you are there? Who is going to manage it? What is the business case? It is a beautiful initiative, but an initiative without a business case.” [P1]	
	15. Dynamic community hub	The guide should serve as a platform for professionals to dynamically contribute and update information		“Imagine, I have a barrier, where else can I add it?” [P11]	
	16. Focus on implementation	Improve the focus on implementation, as it is a crucial component of intervention development		“It is part of its development, but it is also a huge success factor for the use of eHealth, and how you implement it is most certainly different for the low SEP.” [P5]	
**General**					
	17. Enhance credibility	Demonstrating the credibility of the website		“Yes, maybe it could be a little clearer who all this information comes from. Just you as researchers are connected to the university, things like that.” [P2]	

### Quantitative Evaluation of the Prototype

As part of the second interview, the participants evaluated the prototype of the guide across various dimensions (Table 3). All participants completed the questionnaire; however, only the data of 9 out of 10 participants were included in the analysis. The exclusion of 1 participant was due to the questionnaire being modified for clarity and comprehensiveness after receiving feedback from the first participant. Table 3 presents an overview of the participant responses to the questionnaire. In terms of content (eg, barriers and facilitators), participants expressed positive opinions regarding its understandability (89%, 8/9 positive), usefulness (100%, 9/9 positive), and level of interest (100%, 9/9 positive). On the service level, the website was found to be credible (56%, 5/9 positive) and useful (67%, 6/9 positive), and participants would recommend it to a colleague (100%, 9/9 positive). However, most participants did mention they would not want to regularly use the guide (33%, 3/9 positive) since, according to the participants, most of the needed information could be obtained in 1 visit.

**Table 3:** Descriptive statistics of questionnaire responses in the second session (n=9).

Question		Score, mean (SD)	Positive, n (%)	Neutral, n (%)	Negative, n (%)
**General**					
	Try the guide	3.9 (0.3)	8 (89)	1 (11)	0 (0)
**Service**					
	Regularly use the guide	2.7 (1.3)	3 (33)	3 (33)	3 (33)
	Recommend the guide to a colleague	4.0 (0.0)	9 (100)	0 (0)	0 (0)
	Usefulness of the guide	3.4 (1.0)	6 (67)	2 (22)	1 (11)
	Meeting the user’s expectations	2.6 (1.5)	4 (44)	1 (11)	4 (44)
	Credibility of the website and information	3.3 (1.0)	5 (56)	3 (33)	1 (11)
**Content**					
	Usefulness of the barriers and facilitators	4.0 (0.0)	9 (100)	0 (0)	0 (0)
	Interest of the barriers and facilitators	4.0 (0.0)	9 (100)	0 (0)	0 (0)
	Usefulness of the practical stories	3.8 (0.4)	7 (78)	2 (22)	0 (0)
	Clarity of the barriers and facilitators	3.7 (1.0)	8 (89)	0 (0)	1 (11)
	Understandability practical stories	3.7 (1.0)	8 (89)	0 (0)	1 (11)
**System**					
	Ease of use	3.4 (1.1)	6 (67)	2 (22)	1 (11)
	Pleasant to use	3.7 (0.5)	6 (67)	3 (33)	0 (0)

### The Inclusive eHealth Guide

The final product is an interactive web interface (Multimedia Appendix 5). The main component on the website will be used as a starting point, which the user can use as a navigation scheme that shows the phases (eg, development, reach, and adherence) and their corresponding categories (reach strategies and user-friendliness). Using this scheme allows the user to navigate to the category that applies to their specific situation. Within this category, the user will find barriers and their corresponding facilitators. The barriers and facilitators are complemented with practically applicable information such as tips, examples, external tools, resources, and literature. When possible, according to the association scheme of phase 1, user portraits are shown to communicate the users’ perspective. The user portrait consists of an illustration complemented by quotes from the CBPR study. They present a concise and visual representation of key attitudes that provide additional explanation and illustration. Finally, a separate background page provides information about the authors, research team, affiliations, and studies.

## Discussion

### Principal Findings

In this study, we developed an applicable and user-centric guide that supports professionals during the development, adaptation, evaluation, and implementation of eHealth interventions for low-SEP populations. We gained insight into professionals’ needs and preferences regarding system (eg, presentation of information and navigation structure), content (eg, terminology and tone of voice), and service (eg, credibility and viability). Based on these insights, we formulated 16 requirements for the design of the guide. According to these requirements, we developed the final version of the inclusive eHealth guide.

### Feedback on Content and Design

The professionals found the second iteration of the prototype to be beneficial, useful, and usable. We attribute these findings to the integration of their needs and preferences in both its content and design.

On the design level, we found that this second prototype of the website, with its open navigation structure allowing users to switch between general and specific information, was perceived as user-friendly. It is common for researchers to distribute their knowledge through traditional means such as lengthy summaries, reviews, or guidelines [38]. However, these methods can be confusing and frustrating for users. Our guide aims to provide accessible information that can be easily accessed by professionals in the field. In terms of content, professionals appreciated the collection of scientific knowledge and applicable information presented in the prototype. Currently, knowledge about eHealth and people with a low SEP is scattered, making it challenging for professionals to obtain a complete understanding of how to develop eHealth interventions for this target group [23]. Our guide could serve as a centralized hub for acquired knowledge in this area, as it would contain all the necessary information for professionals in one place, taking a significant step in uniting this knowledge and facilitating its wider dissemination.

### Different User Goals

Despite not distinguishing between the various roles of professionals during the interviews, the results underscore the fact that professionals who visit the guide have different goals and needs. This highlights that the existing static materials (eg, summaries, reports, and scientific papers) are not suitable to cater to all professionals’ needs. For example, the developers who participated in this study needed information on various phases (eg, development and evaluation) because they are likely to be involved in different phases of intervention design. This result aligns with previous studies indicating that, within eHealth development, there is a need for flexible and agile development [66]. The health care providers in this study seemed to express less interest in the design process and more in using eHealth and reaching the target group. This is not surprising, as care professionals face many obstacles during the implementation of eHealth interventions due to the lack of explicit training materials and assistance for eHealth users [67]. Some health care providers do not know how to motivate their patients with a low SEP and support them to use eHealth interventions. Swinkels et al [67] conclude that explicit instructions and tips (from other health care professionals) are needed to encourage and persuade patients to use eHealth. The researchers that participated in this study specifically wanted to develop a comprehensive view of the available information to increase awareness of developments in eHealth and vulnerable groups and further organize their research. Researchers in this area mainly use an explorative approach that relies on trial and error [68], making it an uncertain search strategy. As a result, they may not have a clear path for gathering the information. It is notable that policy makers were the least enthusiastic about the current content of the guide. According to the literature, health policy professionals want to make well-informed decisions based on the best available evidence [69]. This may explain why the policy makers in this study valued formal evidence-based information more than user perspectives, indicating that reports and guidelines may better fit their goals than practical information or user perspectives. Understanding the guide users’ goals can help us make the guide more suitable for their needs.

### User Perspective

A noteworthy finding in this research is that professionals acknowledge the significance of incorporating the perspective of the low-SEP target group. Our findings align with the perspectives of Kayser et al [70] and van Velsen et al [71], who emphasize the importance of considering user perspectives, as losing focus on the user perspective can lead to overlooking the needs of the stakeholders. However, we encountered challenges in determining effective ways to represent this user perspective. Our findings suggest that user representations of the low-SEP target group that offer a balance between abstract concepts and realistic representations are preferred over fictional, detailed, and extensive descriptions. Initially, we intended to integrate persona-like user representations on our website, as they are popular tools for communicating user scenarios [72]. Nevertheless, because of the need for short, realistic, and visual representations, personas were not considered the most suitable tool for communicating user perspectives. Instead, we opted for user portraits, which describe a certain user perspective briefly and visually through realistic quotes. The inclusion of user perspectives on the website makes the guide more engaging for the professional and can be a valuable way to share information about the low-SEP group, especially when professionals lack familiarity with this target group. It is important to offer user perspectives because professionals can have different views of low-SEP groups that, at times, do not correspond with reality. Moreover, this approach has the potential to facilitate greater empathy toward this target group.

### Limitations

In this study, we used a broad definition, extending beyond education level, income, and occupation, to describe the low-SEP group. Defining the SEP group is complex because of the high heterogeneity within it, which is why we aimed for broadness to cover not a specific subgroup but to include the lower-SEP group. However, it is important to ensure that the recommendations are indeed applicable to a specific target audience or context. For example, a recommendation that is highly relevant for groups with low health literacy might not be as applicable to groups facing cultural barriers or adverse life events. The guide’s application may vary not only among different low-SEP subgroups but also across patient groups in diverse health care settings and geographical locations. In addition, an argument might be given that using recommendations of the guide, such as simplicity and user-centered design, could also benefit higher-SEP populations. We, therefore, recommend that professionals take additional precautions and consider their specific situation when taking advantage of the different recommendations offered by the guide.

Another important limitation of this research is that we only obtained insights from Dutch professionals. This may decrease the generalizability of the guide to other countries. Hence, the current guide is only available in Dutch. However, it is noteworthy that the challenges faced by, for example, health care professionals in the Netherlands are not unique to this country, as similar barriers are encountered by professionals worldwide [23,73,74]. Therefore, a future step would be to make the guide also available in English.

The content of the guide is based on 2 relatively small-scale studies. Although they provide a valuable perspective by combining insights from both professionals and people with a low SEP themselves, a future step would be to validate the final version of the guide with professionals and to apply the guide in a real-world scenario to learn about its practical applicability in specific contexts.

### Conclusion

In this study, we developed a guide to support professionals during the development, adaptation, evaluation, and implementation of eHealth interventions specifically for low-SEP populations. Through our participatory process, we ensured that the guide aligned with professionals’ needs and preferences and provided information and tools to help them develop appropriate interventions to bridge part of the social health inequality gap between the lower-SEP groups and other groups. Future research should validate the guide to determine its applicability for professionals who want to develop eHealth interventions for people with a low SEP and investigate its practical application in specific scenarios.

## Appendix

Multimedia Appendix 1 Phase 1 Associations between Delphi and community-based participatory research studies.

Multimedia Appendix 2 Prototypes of evaluation sessions.

Multimedia Appendix 3 Interview guides.

Multimedia Appendix 4 Interview detailed results.

Multimedia Appendix 5 Inclusive eHealth guide final version and references to requirements.

## Abbreviations

CBPR: community-based participatory research
IEUQ: Internet evaluation and utility questionnaire
SEP: socioeconomic position
USE: usability, satisfaction, and ease-of-use questionnaire
UTAUT: Unified theory of acceptance and use of technology questionnaire

## Data Availability

The data sets generated and analyzed during the current study are available in the 4TU.ResearchData repository. Access can be requested from the corresponding author.
