# Supplementary material for: Guide Development for eHealth Interventions Targeting People With a Low Socioeconomic Position: Participatory Design Approach
Source: J Med Internet Res. 2023 Dec 4;25:e48461. doi: 10.2196/48461 (PMC10728791; doi:10.2196/48461)
Supplement: Multimedia Appendix 1 [file jmir_v25i1e48461_app1.docx]

# Multimedia Appendix 1: Associations between Study 1 (Delphi Study) and Study 2 (Attitudes Profiles Study)

| Phase | Findings from Study 1 (Delphi Study) | Findings from Study 2 (Attitudes Profiles Study) | Associations between Study 1 and Study 2 |
| --- | --- | --- | --- |
| Development |  |  |  |
|  | B: Knowledge of professionals  (refers to the fact that professionals have insufficient knowledge of people with low SEP) **(-)** | Encumbered  Disadvantaged  Hesitating | - Encumbered: Having a difficult life situation, therefore, thinking about investments for future health has limited priority. - Disadvantaged: Finding written materials too difficult to understand - Hesitating: Having limited digital skills and experiencing difficulties in adopting eHealth. |
|  | F: Social environment  (refers to the involvement of the social environment of people with low SEP in the development of eHealth interventions)  **(+)** | Indifferent  Hesitating | - Indifferent: Has no need for technology but becomes motivated when social networks themselves are enthusiastic about eHealth. - Hesitating: Typically involves people from the social environment with more knowledge and skills to assist in using eHealth. |
|  | F: Rewards  (refer to rewarding the participants for their thoughts on eHealth interventions) **(+)** | Indifferent  Encumbered | - Indifferent: Is not open to nor interested in the development of eHealth. - Encumbered: Becomes motivated by eHealth that relieves burdens or is seamlessly integrated into daily life. |
| Reach |  |  |  |
|  | B: Communication (refers to the verbal and written communication level that does not match with people with low SEP and is therefore unable to reach them) **(-)** | Disadvantaged | - Written materials are too difficult to understand |
|  | B: Lack of resources (refers to lack of time and financial resources to reach the target group) **(-)** | Detached | - Has the lack of trust towards healthcare professionals and researchers. |
|  | B: Engagement (refers to current eHealth interventions that do not sufficiently motivate or stimulate the user ) **(-)** | Eager | - Becomes motivated by self-monitoring and feedback |
|  | B: Everyday life (refers to eHealth interventions that do not align with the everyday lives of people with low SEP) **(-)** | Encumbered | - Has a difficult life situation and, therefore, thinking about investments for future health has limited priority |
|  | B: Modes of delivery (refers to materials and technology that do not fit the skill levels of people with low SEP)  **(-)** | Disadvantaged  Encumbered | - Disadvantaged: Values a healthcare provider that understands communication barriers, listens well, and can explain things clearly. - Encumbered: Values a healthcare provider who understands and considers their life situation. |
|  | B: Usability (refers to the barriers that affect the user-friendliness of eHealth interventions, thus hindering reach to the low SEP group) | Hesitating  Indifferent | - Hesitating: Has limited digital skills and, therefore, experiences difficulties adopting eHealth. - Indifferent: Does not see why eHealth is better than traditional approaches and, therefore, considers it not worth the effort. |
|  | F: Reward (refers to rewarding people with low SEP to encourage participants) | Encumbered  Indifferent | - Encumbered: Becomes motivated by eHealth that deburdens or is seamlessly integrated into daily life. - Indifferent: Does not see why eHealth is better than traditional approaches and therefore considers it not worth the effort. |
|  | F: Communication (refers to different forms of communication) **(+)** | Disadvantaged  Loyal  Eager  Hesitating | - Disadvantaged: 1. Written materials are too difficult to understand. 2. Becomes motivated by visual information that attracts attention. - Loyal: Values a personal, face-to-face approach with (healthcare) professionals. - Hesitant: Is not sufficiently aware of eHealth and what it adds and therefore finds it hard to understand the added benefit of using eHealth. - Eager: Is open towards and enthusiastic about eHealth. |
|  | F: Knowledge of professionals (refers to professionals having the necessary communication skills to reach people with low SEP) **(+)** | Disadvantaged | - Values a healthcare provider that understands communication barriers, listens well, and can explain things clearly. |
|  | F: Everyday life (refers to understanding the everyday lives of people with a low SEP to devise a suitable intervention) **(+)** | Light-hearted/Concerned  Encumbered | - Light-hearted: Is positive and feels good about personal health. Concerned: Faces one or several health-related limitations. - Encumbered: Becomes motivated by eHealth that deburdens or is seamlessly integrated into daily life. |
|  | F: Motivation (refers to motivating people with low SEP about the relevance of eHealth interventions to enhance reach) **(+)** | Hesitating  Indifferent | - Hesitating: Is not sufficiently aware of eHealth and what it adds, and therefore finds it hard to understand the added benefits of using eHealth. - Indifferent: Is not open to nor interested in eHealth. |
|  | F: Reach Strategies (refers to strategies that can be used to reach people with low SEP) **(+)** | Indifferent  Hesitating | - Indifferent: Is not open to nor interested in eHealth. - Hesitating: Is not sufficiently aware of eHealth and what it adds, and therefore finds it hard to understand the added benefits of using eHealth. |
|  | F: Social environment (refers to the social environment of people with low SEP) **(+)** | Concerned  Indifferent  Hesitating  Encumbered  Detached | - Concerned: 1. Has a low perceived control 2. Benefits from social interaction. - Indifferent: Does not see why eHealth is better than traditional approaches and therefore considers it not worth the effort. - Hesitating: In this profile, family members or friends have more knowledge about technology compared to people with low SES. - Encumbered: Benefits from social interaction. |
|  | F: Technology Support (refers to helping people with low SEP in the use of eHealth interventions to enhance reach) **(+)** | Hesitating | - Hesitating: Has limited digital skills and therefore experiences difficulties adopting eHealth. |
|  | F: Usability (refers to the factors that promote the user-friendliness of eHealth interventions) **(+)** | Disadvantaged  Eager  Disadvantaged  Hesitating | - Disadvantaged: Becomes motivated by visual information that attracts attention. - Disadvantaged: Written materials are too difficult to understand. - Eager: Becomes motivated by self-monitoring and feedback. - Hesitating: Is not sufficiently aware of eHealth and what it adds, and therefore finds it hard to understand the added benefits of using eHealth. |
| Adherence |  |  |  |
|  | B: Knowledge (refers to the understanding that individuals with low (SEP) have about their health) **(-)** | Encumbered | - Has a difficult life situation, and therefore, thinking about investments for future health has limited priority. |
|  | B: Motivation (refers to the level of motivation that individuals with low SEP have to continue utilizing interventions) **(-)** | Indifferent | - Does not see why eHealth is better than traditional approaches and therefore considers it not worth the effort. |
|  | B: Not involving people with low SEP (refer to the expectations that individuals of low SEP have towards eHealth interventions and the needs they have in return) **(-)** | Encumbered | - Has a difficult life situation, and therefore, thinking about investments for future health has limited priority. |
|  | B: Usability (refers to the barriers that prevent individuals with low digital skills and low literacy from effectively using eHealth interventions) **(-)** | Hesitating | - Has limited digital skills and therefore experiences difficulties adopting eHealth. |
|  | F: Communication (refers to the means of communicating with individuals with low SEP) **(+)** | Encumbered  Loyal  Concerned | - Encumbered: Has a difficult life situation, and therefore, thinking about investments for future health has limited priority. - Loyal: Values a personal, face-to-face approach with healthcare professionals. - Concerned: Has a low perceived control. |
|  | F: Social environment (refers to involving friends, family, and community support in the utilization of eHealth interventions) **(+)** | Concerned  Encumbered  Hesitating | - Concerned: Benefits from social interaction. - Hesitating: Typically involves people from the social environment with more knowledge and skills to assist using e-health. |
|  | F: Engagement eHealth (refers to the level of involvement and active participation of users in utilizing eHealth technologies) **(+)** | Indifferent/Encumbered  Hesitating/Eager/Indifferent  Encumbered/Concerned/Indifferent  Concerned  Loyal | - Encumbered: Has a difficult life situation, and therefore, thinking about investments for future health has limited priority. - Indifferent: Is not open to nor interested in eHealth. - Hesitating/Eager/Indifferent - Hesitating: Is not sufficiently aware of eHealth and what it adds, and therefore finds it hard to understand the added benefit of using eHealth. - Eager: Becomes motivated by self-monitoring and feedback. - Indifferent: Does not see why eHealth is better than traditional approaches and therefore considers it not worth the effort. - Concerned: 1. Has a low perceived control. 2. Becomes motivated by setting and achieving goals. - Loyal: Values a personal, face-to-face approach with healthcare professionals. |
|  | F: Usability (refers to enabling individuals with low SEP, low digital skills, and low literacy to access and use eHealth interventions effectively) **(+)** | Hesitating | - Has limited digital skills and therefore experiences difficulties adopting eHealth. |
| Evaluation |  |  |  |
|  | B: Evaluation methods and timing (refers to the evaluation methods used during evaluation research that do not fit the low SEP group) **(-)** | Disadvantaged | - Disadvantaged: Written materials are too difficult to understand. |
|  | B: Everyday life (refers to people with low SEP who have other problems that prevent them from participating in evaluation research)  **(-)** | Encumbered  Concerned | - Concerned: Faces one or several health-related limitations. - Encumbered: Has a difficult life situation and, therefore, thinking about investments for (future) health has limited priority. |
|  | B: Engagement (Refers to methods or strategies that encourage people with low SEP to participate in evaluation studies) **(-)** | Encumbered  Concerned  Indifferent  Detached /Loyal | - Encumbered: 1. Has a difficult life situation and, therefore, is not eager to participate in evaluation studies. 2. Has a difficult life situation and, therefore, thinking about investments for (future) health has limited priority. - Concerned: Becomes motivated by setting and achieving goals. - Indifferent: Does not see why eHealth is better than traditional approaches and, therefore, considers it not worth the effort. - Detached: Has a lack of trust towards healthcare professionals and researchers. - Loyal: Values a personal, face-to-face approach with (healthcare) professionals. |
|  | F: Evaluation methods (refers to the evaluation methods used to evaluate the eHealth intervention) **(+)** | Disadvantaged | - Disadvantaged: Written materials are too difficult to understand. |
| Implementation |  |  |  |
|  | F: Motivation (refers to persuading professionals to implement an eHealth intervention in practice) **(+)** | Hesitating | - Hesitating: If professionals themselves are competent and enthusiastic about eHealth, it can be effective in hesitant groups |

*B* barriers, *F* facilitators
