# Supplementary material for: Guide Development for eHealth Interventions Targeting People With a Low Socioeconomic Position: Participatory Design Approach
Source: J Med Internet Res. 2023 Dec 4;25:e48461. doi: 10.2196/48461 (PMC10728791; doi:10.2196/48461)

## Multimedia Appendix 2 – Prototypes

### Evaluation session 1 - Low fidelity prototypes


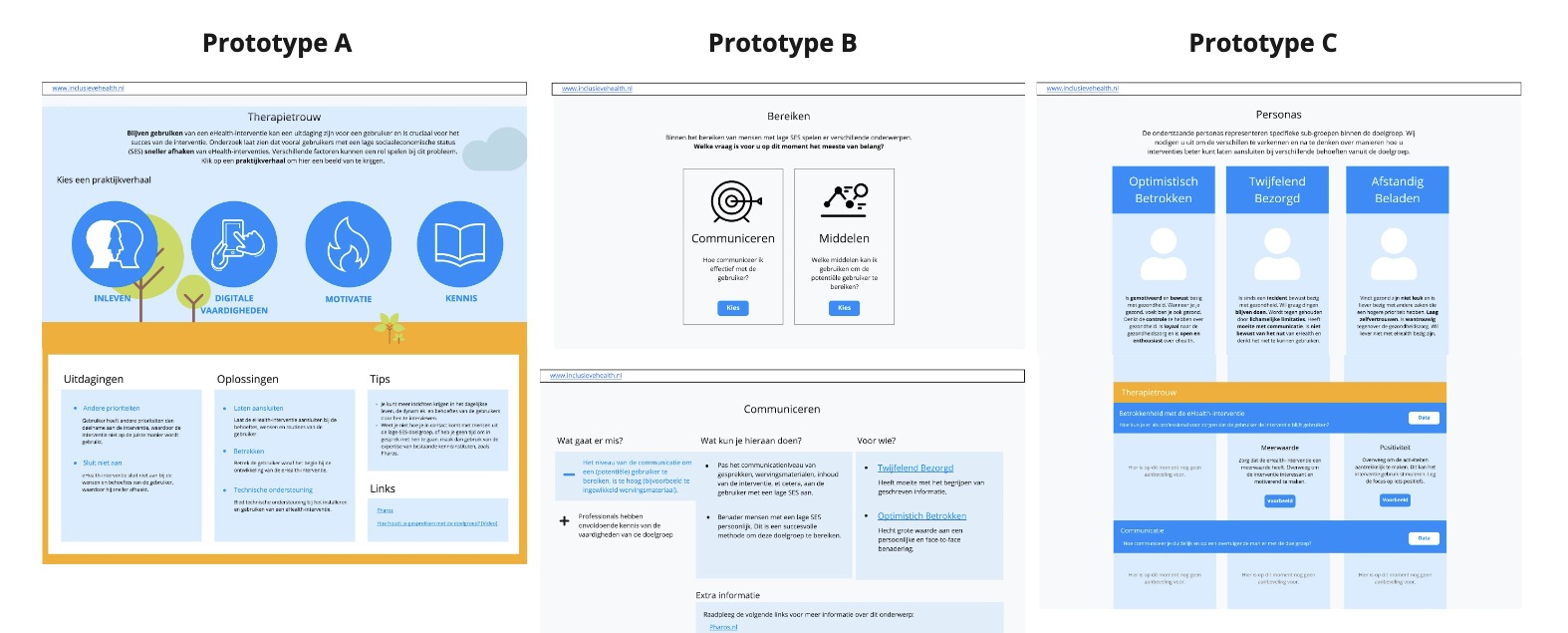


### Evaluation session 2 – High fidelity prototype
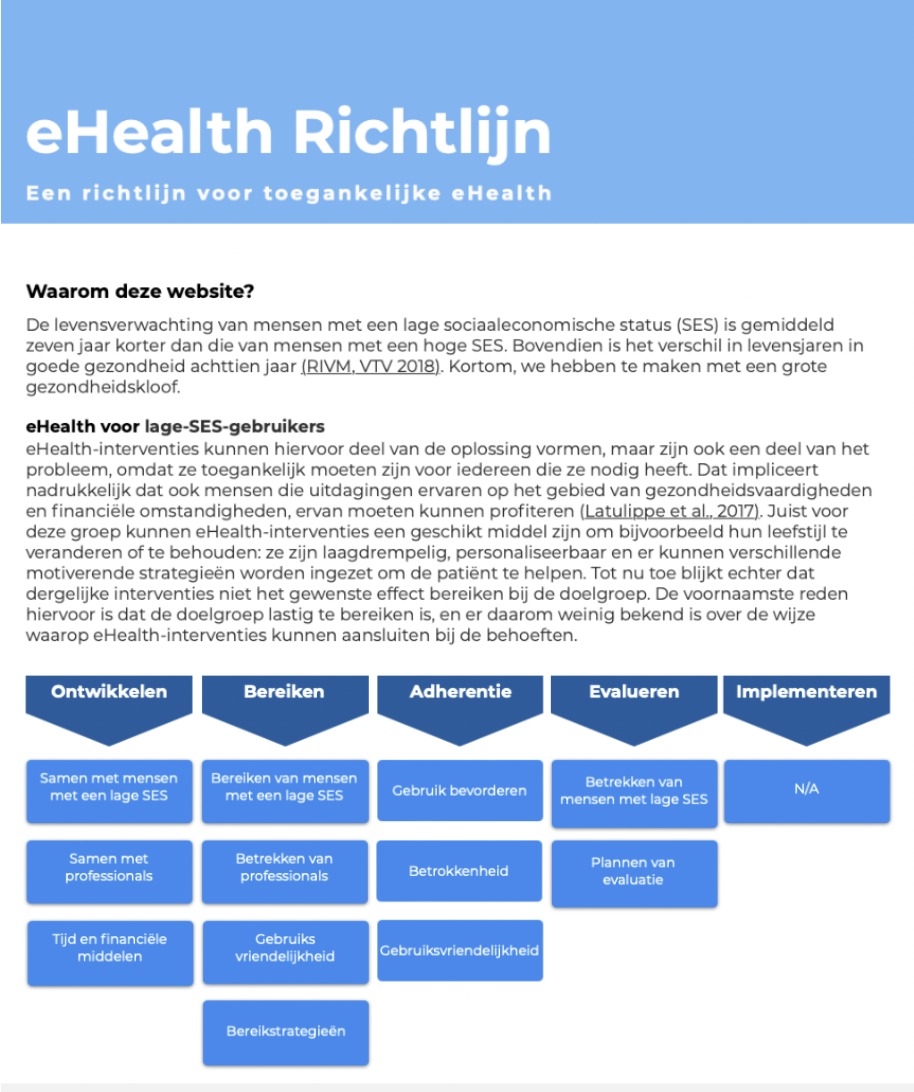


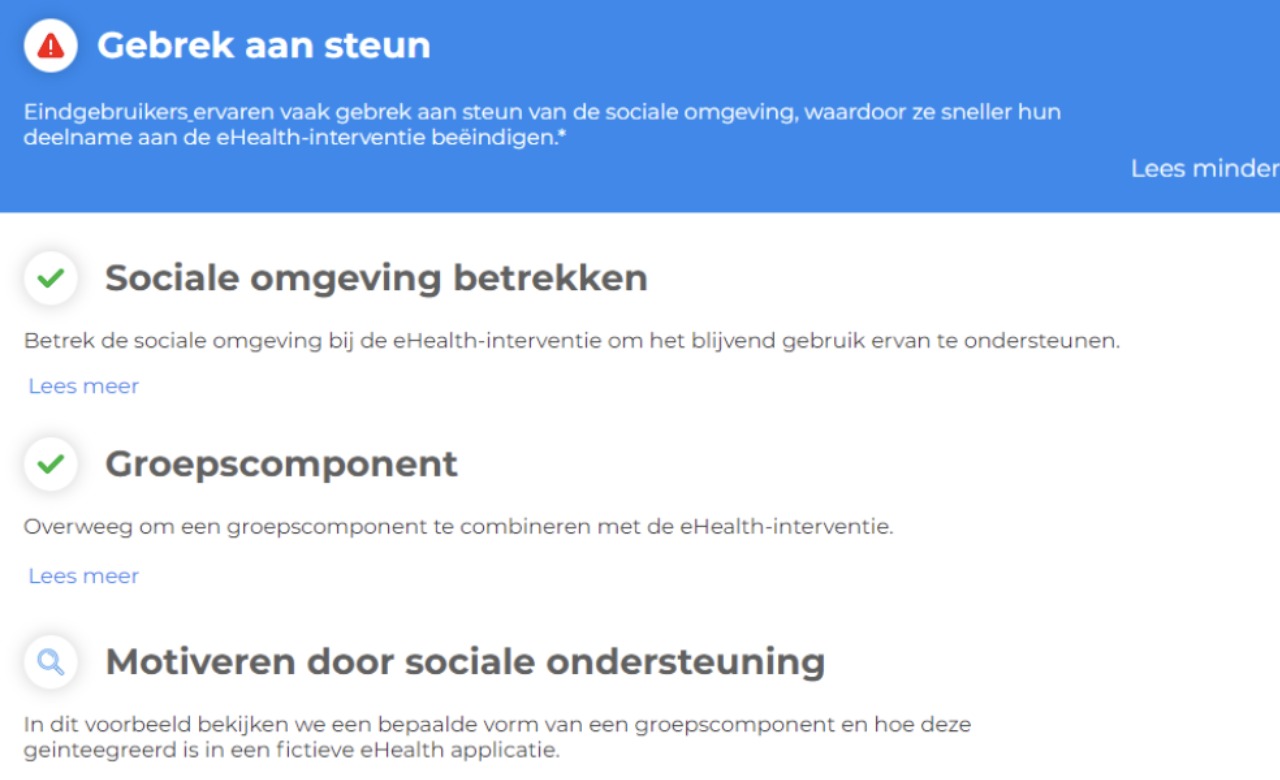

Supplement: Multimedia Appendix 2 [file jmir_v25i1e48461_app2.docx]
