# Supplementary material for: Guide Development for eHealth Interventions Targeting People With a Low Socioeconomic Position: Participatory Design Approach
Source: J Med Internet Res. 2023 Dec 4;25:e48461. doi: 10.2196/48461 (PMC10728791; doi:10.2196/48461)
Supplement: Multimedia Appendix 3 [file jmir_v25i1e48461_app3.docx]

## Multimedia Appendix 3 – Interview guides

### Interview guide session 1

**Part 1: Background questions**

- What is your current position?
- Do you work professionally in improving the health of people with low socioeconomic position (SEP)? If yes, how are you involved with people with low SEP through your work?
- Developing or adapting (eHealth) interventions
- Applying (eHealth) interventions to people with low SEP
- Policy development
- Scientific research
- Working in healthcare
- Other, namely ………
- Which eHealth applications do you use?

**Part 2: Prototypes**

First, we will provide you with a fictional scenario of a potential website visitor. Then, we will show you three different test versions of the guideline. Each test version consists of a number of screens. While you navigate through the different screens, we ask you to describe out loud what is going on in your head. The goal is for us to get the best possible idea of what you expect from a website and how well our test versions meet those expectations. In short, we want to hear your opinion. It is important to know that we are testing the test versions and not you. So, don't worry: you cannot make mistakes or hurt our feelings. We are conducting this research to improve the test versions, so we want to hear honest feedback. Is the explanation clear so far? If yes, the researcher will start the test. If you need a break, please let us know.

Introduction

The target group for which we built this website 'eHealth guideline' consists of experts (such as healthcare providers, researchers, policymakers, developers) who want to develop or adapt eHealth interventions for users with low socioeconomic position (SEP). The aim of this website is to provide information and practical support to experts if they want to know more about how to develop or adapt eHealth interventions for users with low SEP.

Scenario’s

| Scenario | Developer | Healthcare worker | Researcher |
| --- | --- | --- | --- |
| Current situation | You want to redevelop an existing app that helps with smoking cessation so that it better meets the needs of people with a low socioeconomic position. | You want to know which online intervention or app is best to use in your practice. You deal with many people with a low socioeconomic position and want to know which apps work well specifically for this target group. | You are involved in research on making eHealth suitable for people with a low socioeconomic position. You are curious about what is currently known and what you can learn from it. |
| Problem | You are not sure what to take into account and have little time to do extensive research on this. | You think it is hopeless and that apps are not suitable for your target group. You do not know if there are any possibilities. | There is a lot of information available on various websites and papers. You are looking for a central place for all this information. |
| Discovery | A colleague tells you about an online guideline for developing eHealth interventions for people with a low socioeconomic position. You decide to visit the website. | | |
| Goal/question | Your goal is to quickly get an idea of what to expect. This will give you a starting point from which you can continue your work. | Your goal is to find out what the possibilities are. With this knowledge you can continue to search for the right apps. | Your goal is to quickly get a good overview of the information and to quickly access the source of the information via the website. |

Prototype 1

Frame 1 – Homepage. You arrive at the website. What do you see here? What would you do now? What do you expect to see if you do that? You click on continue. Explain what it is intended for.

Frame 2 - Choose a phase goal. What do you see here? What would you do now? What do you expect if you do that? You choose the goal of therapy adherence.

Frame 3 - Choose a situation. What do you see here? What would you do now? What do you expect if you do that? You choose to explore the situation further.

Frame 4 - Read the situation. What do you see here? What do you think of this information? What would you do with this information? What would you do now? What do you expect if you do that? You choose to explore the explanation for this situation further.

Frame 5 - Read the explanation. What do you see here? What do you think of this information? What would you do with this information? What would you do now? This was the end of the first prototype.

Interim evaluation

- What did you think of this prototype?
- Can you name two strong points and two weak points? And indicate why?
- What did you think of the way you could navigate the website?
- What did you think of the way the information within the website was structured?
- What did you think of the information on the website?
- What did you think of the way the information was presented?
- If you could change anything about the information provided, what would it be?
- What was easy or difficult to understand?

Prototype 2

Frame 1 – Homepage. You arrive on the website. What do you see here? What would you do now? What do you expect to see when you do that? You click on "continue".

Frame 2 – Choose a goal or persona. What do you see here? What would you do now? What do you expect to see when you do that? You now choose a goal or user. Which one would you choose?

Frame 3.1 – Choose a theme. What do you see here? What would you do now? What do you expect to see when you do that? You choose to explore theme 1 further.

Frame 3.2 – Read persona. What do you see here? What do you think of this information? What would you do with it? What would you do now? What do you expect to see when you do that? You choose to explore a related theme further.

Frame 4 – Read theme. What do you see here? What do you think of this information? What would you do with it? What would you do now? This was the end of the second prototype.

Interim evaluation

- What did you think of this prototype?
- Can you name two strong points and two weak points? And indicate why?
- What did you think of the way you could navigate the website?
- What did you think of the way the information within the website was structured?
- What did you think of the information on the website?
- What did you think of the way the information was presented?
- If you could change anything about the information provided, what would it be?
- What was easy or difficult to understand?

Prototype 3

Frame 1 – Homepage. You arrive on the website. What do you see here? What would you do now? What do you expect to see when you do that? You click on "continue".

Frame 2 – Explore personas. What do you see here? What would you do now? What do you expect to see when you do that? You now scroll down to read more about the different personas.

Frame 3 – Choose data. What do you see here? What would you do now? What do you expect to see when you do that? You choose to explore the data for...

Frame 4 – Read data What do you see here? What do you think of this information? What would you do with it? What would you do now? What do you expect to see when you do that? You choose to go back and view an example.

Frame 4 – Read example (screen with the "ommetje" app). What do you see here? What do you think of this information? What would you do with it? What would you do now? This was the end of the second prototype.

Interim evaluation

- What did you think of this prototype?
- Can you name two strong points and two weak points? And indicate why?
- What did you think of the way you could navigate the website?
- What did you think of the way the information within the website was structured?
- What did you think of the information on the website?
- What did you think of the way the information was presented?
- If you could change anything about the information provided, what would it be?
- What was easy or difficult to understand?

**Closing remarks**

We have come to the end of the developed evaluation.

- Which of the prototypes (1, 2, or 3) appealed to you the most? Can you explain why you prefer (1, 2, or 3)? Which of the prototypes (1, 2, or 3) appealed to you the least? Can you explain why you prefer (1, 2, or 3)? Why?
- Are there any other aspects you would like to address?

### Interview guide session 2

**Introduction**

- What is your current position?
- Do you work professionally in improving the health of people with a low socioeconomic position (SEP)? If yes, ... In what way are you involved with people with a low SEP through your work?
- Developing or adapting (eHealth) interventions
- Applying (eHealth) interventions to people with a low SEP
- Policy development
- Scientific research
- Working in healthcare
- Other, namely ……
- What eHealth applications do you work with?

**Part 1: Think Aloud**

In this section, you will perform a task while thinking aloud. It may feel unfamiliar and uncomfortable to think aloud. It is important to know that this is not a test for you but for the prototype. Try to describe what you are doing and why at each step you take. For example, if you enter a search term on Google, you can say, "I am searching for eHealth, I type it into the address bar, and I hit enter. Now, I see all kinds of websites related to my search term. I am scanning through them. This website looks interesting, etc." We will start with a scenario, a situation in which you find yourself. Then, you will receive a set of instructions that you must follow while thinking aloud. Is the explanation clear so far? (If yes, the researcher will start the test.) If you want to take a break, you can let us know.

Scenario

You are designing an app to motivate people with a low SEP to adopt a healthy lifestyle. You hear about this website from a colleague and wonder if the website can provide you with some guidance to make the app more suitable for people with a low SEP.

Start

- In case of online: You can now find the link to the website in the chat. Can you go there and share your screen with us?
- Task 0: Take a few minutes to look around the site. Let us know when you're done.
- Task 1: Read the introduction text on the first page at your own pace. When you're done, can you give us a summary of what you've read?
- Task 2: You're curious about the challenges involved in promoting the use of an e-health intervention by low SEP users. Go to the hindering factors regarding the use of an e-health intervention.
- Task 3: One challenge stands out for you: Long-term goals. This is often cited as a difficult problem in your field. You're curious about what you can do about it.
- Go to the promoting factor focused on long-term goals.
- Task 4: You're wondering how this could be implemented in an app form. Go to how short-term goals can be integrated into an app.
- Task 5: Lastly, you're wondering why it's important for people with low SEP to break down big goals. Find out why it's important for someone with low SEP to set short-term goals.

**Part 2: Questionnaire**

Thank you for thinking out loud, that gives us more insight into where the areas for improvement lie. Now we would like to ask you a few questions about your impression of the guideline on various aspects, such as relevance, reliability, and user-friendliness. Then we will further explore the reason. Assuming that the website is fully completed, I will first ask you general questions (usability and intention), then questions about the content of the website, and finally about the design.

*Intention (General)*

- Would you like to try the guideline?
- Would you like to use the guideline on a regular basis?
- Would you recommend the guideline to a colleague?
- Usability (General)
- Do you think the guideline is usable?
- Does the guideline do everything you expected it to do?

Before we move on to the next questions, I would like to ask you to consider 1 or 2 barriers or facilitators.

*Relevance (Content)*

Do you find the barriers and facilitators useful?

Do you find the barriers and facilitators factors interesting?

Do you find the practical stories useful?

*Comprehensibility (Content)*

- Do you find the barriers and facilitators clear?
- Do you find the practical examples easy to understand?

*Reliability (Content)*

- Did you feel that you could trust the information?
- User-friendliness (Design) Do you find the guideline easy to use?

*Satisfaction (Design)*

- Do you find the guideline pleasant to use?

**Part 3: Open Questions**

Finally, we would also like to ask you a few open questions. These questions are intended to gather additional information that we did not cover in the previous sections.

- What is your overall impression of the guideline?
- What was the most useful part of the website?
- What was the least useful part of the website?
