# Supplementary material for: Guide Development for eHealth Interventions Targeting People With a Low Socioeconomic Position: Participatory Design Approach
Source: J Med Internet Res. 2023 Dec 4;25:e48461. doi: 10.2196/48461 (PMC10728791; doi:10.2196/48461)
Supplement: Multimedia Appendix 4 [file jmir_v25i1e48461_app4.docx]

# Multimedia Appendix 4 – Interview detailed results

## Interview 1 detailed results

### System

Based on our analysis we divided the resulting themes regarding the system into two categories: navigation and presentation. Regarding navigation, we found preferred navigation pathways. The majority (n = 5) of the participants indicated they preferred a *convergent* navigation strategy, starting with broad and general information and tapering into more specific and detailed information. Five participants acknowledged a need for *open* navigation, which they explained as being able to reach the desired information through multiple pathways instead of a predetermined (closed) navigation route. Five participants commented on the *information structure*. They, for example, described the value of the *neutral* prototype, which made a distinction between development phases (e.g., reach, adherence, evaluation etc.) and categories within these phases. Regarding the presentation of the prototypes, three participants needed more *structure* in the *presentation*. Two remarked the importance of *more* *visuals* while two others indicated some of the current visuals were *confusing.*

### Content

We discovered several themes related to the *depth of information*, *practice*, *terminology*, *tone of voice* and *credibility*. For *depth of information,* all participants indicated the need for *applicable information*. They expressed that the information should address not only *what* is needed or important, but also *how* it should be applied in practice. In addition, five participants questioned the information’s usefulness as in some cases it was not *low SEP specific.* Three participants had issues with the comprehensibility of the information. Regarding the practice*,* most (n = 5) participants saw the value of using *personas*. Three participants favored the practical side of the personas to complement the theoretical barriers and facilitators, though two participants questioned the credibility of some of these practical suggestions. We found mixed reactions regarding the clarity of the *terminology*. While three participants thought the *terminology was clear*, two others were uncertain about the terms used (e.g., specificity). In addition, three participants warned to *avoid jargon* and technical terms as much as possible. Regarding the *tone of voice,* we found four participants commenting on the *way they should be addressed;* participants showed preference for being addressed in a more informal manner. Three participants demonstrated that aspects of the guide’s content were sometimes too *negatively framed* (e.g., we have insufficient attention for your user).

### Service

The service category themes related to the *goal* of the guide (what is the reason for users to visit) and addressed the more *general* topics of the guide. Regarding the goal of the guide, most participants indicated they would like to use the guide to *explore* available information (n = 5), *judge* the alignment of their intervention with the recommendations (n = 6), *collect knowledge* (n = 5) and practically *applicable information* (n = 5). The participants also indicated they would expect to be *inspired* by already existing initiatives and examples (n = 3) and *empathize* with the target group (n = 3). Regarding the general guide topics, almost all participants (n = 6) indicated they saw value in *referencing* and directing visitors to other, already existing, sources of information. Five participants stressed the importance of *user tailoring* the focus of the website towards different user groups (e.g., developers and healthcare practitioners). Three participants stressed the importance of *viability* of the guide. They expressed concerns regarding the maintenance of the guide after its initial release. Five participants indicated the importance of information being *evidence-based.* They stressed the need for all information provided being backed-up by (scientific) evidence.

Interview 1: Descriptions, codes, and quotes per theme and category

| **Theme** | | **Type** | **F** | **Description** | **Quote** |  |
| --- | --- | --- | --- | --- | --- | --- |
| **Service** | | | | | | |
| **Category: Goals** | | | | | | |
|  | |  |  |  |  |  |
| Apply | | N | 15 | Wanting to have practical information and examples. | *“I was curious about: when are you doing well? I would like to have more good examples.”* (P5) |  |
| Collect | | N | 11 | Wanting to collect useful information for the project. | *“I would start with looking for hands on tools and practical knowledge that is already available that I can use to design an eHealth module.”* (P4) |  |
| Judge | | N | 10 | Wanting to be able to judge the effectiveness of existing research or applications | *“I want to validate whether the research, or a certain eHealth application is effective. [...] If have to wonder on a lot of aspects whether it is effective for low SEP.“ (*P3) |  |
| Explore | | N | 10 | Wanting to have an overview the knowledge that is available. | *“It never is fully complete, but I would like to have an overview of what are all the applications that are specifically aimed at people with a low SEP?”* (P2) |  |
| Empathize | | N | 8 | Wanting to have insight into the target group. | *“I would want to have more insight into the target group. What is the target group, what are its characteristics? What are personas?”* (P1) |  |
| Inspire | | N | 5 | Wanting to be inspired by already existing initiatives. | *“I think it is important to provide some inspiration from what other people already researched. So, you don't have to reinvent the wheel”* (P1) |  |
| Dynamic goals | | N | 3 | Wanting the focus of the website to adapt to the changing needs | *“If it was two years ago there would have been other needs”* (P1) |  |
| **Category: General** | |  |  |  |  |  |
|  | |  |  |  |  |  |
| Viability | | R | 16 | The website should be viable and be supported after its initial release. | *“This is one of the most challenging aspects; you can make a beautiful website, but who is going to visit it? Who knows you are there? Who is going to manage it? What is the business case? It is a beautiful initiative, but an initiative without a business case.”* (P1) |  |
| User tailoring | | R | 11 | The need to appeal to the needs of different user groups. | *“Well I do think that it would appeal to healthcare practitioners towards people they see in their daily practice.”* (P7) |  |
| Referencing | | R | 10 | The guide being a starting point referencing to other sources instead of containing all the information itself. | *“The proper references. You wouldn't want to be the place, like wikipedia, where you can find all the information. No, I think that an important function is referencing.”* (P1) |  |
| Credibility | | R | 7 | The need to see evidence on which the claims are made | *“I would like to trust that what is said, is good. That it is evidence based. Based on scientific evidence.”* (P1) |  |
| Stratification | | Ne | 7 | The difficulty of stratifying user groups and problems into categories. | *“You are going to think about what small part of the problem it is but, it is actually more general. So, the in-between step is maybe not so useful.”* (P5) |  |
| Broad spectrum | | N | 3 | The subject encompasses many aspects. | *"Unfortunately, it is a very broad concept and you will have to add many things if you want to create value.”* (P1) |  |
| **Content** | | | | | | |
| **Category: Information** | | | | | | |
|  |  | | | | |  |
| Applicability | | R | 40 | The need to know how to apply the information in practice. | *‘’I would have expected more guidance, say I visit the website and want to adapt something, how should I do it?’’* (P2) |  |
| Credibility | | R | 11 | The information provided being based on evidence | “That I can trace back: Where does it come from? Where is it based on?” (P6) |  |
| Low SEP specific | | R | 10 | Wanting information that is specifically aimed at people with a low SEP. | *“You share three core insights, but these are not specifically focussed on people with a low SEP?”* (P1) |  |
| Comprehensibility | | Ne | 7 | The ability to properly understand the provided information | *“For the attitude part it was not directly clear for me that you were talking about the patient, till I started reading.”* (P1) |  |
| **Category: Tone of Voice** | | | | | | |
|  |  | | | | |  |
| Way of addressing | | N | 8 | The way in which the user of the guidel wants to be addressed | *“I do think it is more personal. Especially when you are a developer. I would do that. That is my taste, but I wouldn't care if I was to be addressed more formally.”* (P5) |  |
| Negative framing | | Ne | 6 | Some terms in the guide are framed too negative | “I'm always very cautious with negative terms myself; "we have insiffucient attention" That is a very deniying term and is not very positive, I must say.” (P3) |  |
| Personal | | R | 3 | Preferring to be addressed in a personal way | *“I can imagine that in the last one, [...] that being addressed in a personal way could work. But i'm not sure, it is a bit of choice, the style you choose for.”* (P3) |  |
| **Category: Terminology** | | | | | | |
|  | |  |  |  |  |  |
| Avoid Jargon | | R | 7 | The avoidance of technical terms and jargon. | *“Tunneling, I don't think that is a term that directly calls to someone.”* (P3) |  |
| Terminology is clear | | P | 4 | Terms used in the guide are clear | *“Technical support I totally understand, the terminology you use helps with that.”* (P3) |  |
| Terminology is not clear | | Ne | 4 | Terms used in the guide are not clear | *“Resources might also be categorized as communication. So, you could make it more specific.”* (P5) |  |
| Languages | | Ne | 3 | Issues related to the used language | *“Because inclusive health is a mix of Dutch and English so it’s difficult for the user to remember that.” (P5)* |  |
| **Category: Practice** | | | | | | |
|  |  | | | | |  |
| Persona preference | | P | 7 | Being positive about the use of personas | *“On the one hand it will clarify the context of your barriers and facilitators. So, I’m generally a big fan of personas”* (P5) |  |
| Practical examples | | R | 4 | Favoring practical examples to complement theoretical information. | *“I do like option B (prototype) because it is neutral. But I do miss the practical side of it. The practical examples are very useful.”* (P2) |  |
| User stratification | | Ne | 4 | The difficulty to stratify user groups. | *“You can never have too many personas to encompass everyone.”* (P5) |  |
| **System** | | | | | | |
| **Category: Navigation** | | | | | | |
|  |  | | | | |  |
| Persona-based | | R | 13 | Not wanting to use a persona as a search strategy, but rather as a dimension to specify information | *“The example of personas is of crucial importance; however, a persona is just a dimension.”* (P1) |  |
| Convergent | | P | 12 | Wanting to navigate from generic to more specific information | *“It feels like a nice step, you have two new categories within a category. So, you are talking about reach, and you can see for yourself which aspect of reach is important to me.”* (P4) |  |
| Information structure | | N | 7 | Comments regarding the way the content is structured. | P7: I like that there is a structure between challenges, solutions, and attitudes. I think that people, care providers, could benefit from them. |  |
| Explanation | | Ne | 6 | The need for navigation instruction | *“You have to provide a bit more information”* (P2) |  |
| Open | | R | 5 | Doesn't want to be directed through a set navigation pathway | *“In terms of user experience, I would like to navigate through different pathways. That would be my most important requirement.”* (P5) |  |
| Phase-based | | P | 4 | Prefers to navigate using phases of development | *“The structure is very logical. It is exactly as i said; I expected these are the steps you must make as a developer or the phases.”* (P5) |  |
| Burden | | Ne | 4 | Avoiding that the user must go through a lot of information to get where they need to be. | *“It is a lot of clicking. So, what is the added benefit? I must go through to the next, scrolling for a while and click through.”* (P2) |  |
| Goal-based | | P | 3 | Preferring to search information based on goals | *“Through this I can easily and goal-orientedly choose and navigate the information I would like to have”* (P1) |  |
| Quick | | R | 3 | Wanting to find information quick | “Actually, you want to find information as quickly as possible.” (P2) |  |
| **Category: Presentation** | | | | | | |
|  |  | | | | |  |
| Presentation structure | | Ne | 7 | The need for more structure within the presentation of the information | *“That part with ‘tips’ feels quite cluttered, also because of the blue headers and blue hyperlinks.”* (P2) |  |
| More visuals | | R | 3 | The need for use of more visuals and icons | *“Indeed, when you use fun images, with a name and large format, people will become curious automatically.”* (P6) |  |
| Confusing visuals | | Ne | 3 | Some of the current visuals are perceived as confusing | *“Because of the trees in the background, it feels a bit crowded and because of that I’m less drawn to the image.”* (P7) |  |

(N = Neutral, R = Future recommendation, Ne = Negative, P = Positive, F = frequency of code)

## Interview 2 detailed results

### System

Almost all participants (n = 8) suggested more positively *ordering the barriers and facilitators*. Four participants recommended *beginning with facilitators* instead of barriers. Six participants found the barriers and facilitators pages *visually unappealing* because they lacked images. Five participants recommended that these pages incorporate *visual elements*.

Four participants found the stories *layout unappealing*, partly because the text was not clearly visible. The *visual elements* (e.g., images and apps) in the stories page were experienced positively by the participants (n = 3). Two participants recommended using *videos* to tell the stories of low-SEP groups rather than just text.

There was a positive response from the participants (n = 7) to the scheme with the recommendations and themes. Consequently, most participants (n = 8) believed this scheme should be used for *onboarding*. Participants found the website to be visually *unappealing*. They recommended “improving the visual appeal” of the website through color and images. Forty percent of the participants felt that the navigation of the website requires too much effort (e.g. scrolling and clicking). The recommendation (n = 5) was, therefore, to provide “easier navigation.”

### Content

Most participants (n = 6) found that the barriers and facilitators contained *interesting content*. Six participants also expressed a positive view of the *credibility* of the barriers and facilitators. However, most participants (n = 6) stated that they need *applicable information*. Nonetheless, several participants (n = 4) indicated that the *tone of voice* used for the barriers and facilitators could be improved. For example, by changing the suggestiveness of the content to more concrete advice. The majority of participants (n = 8) found that the stories provide the opportunity to *empathize with the low-SEP* group and are *helpful*. Some participants (n = 6) felt that *real stories* should be used instead of hypothetical ones and suggested making them more concrete (applicability). Additionally, participants suggested that the guide should *expand the stories* to include different variants of people with a low SEP. Most of the participants (n = 8) believed the website contains *too much text*. Consequently, seven participants recommended *conciseness* texts to increase readability. Fifty percent of participants also stressed the importance of including information about implementation (importance of implementation).

### Service

In general, participants (n = 6) appreciated the references used to support the information provided. In addition, participants (n = 5) found the website lacking in credibility (credibility issues). As a result, seven participants suggested using logos and the contact information of the people behind the website to demonstrate *credibility*. Participants suggested using the website as a starting point for eHealth interventions for this target group, as they find it helpful for considering relevant factors and using it as a knowledge community. In addition, they would recommend it to others (e.g., collaboration partners). According to the participants (n = 7), the website should be customized to meet the needs of different users (User-profile tailoring). Since the information included on the website is informative, the term “guide” should be used, according to six participants.

Interview 2: Descriptions, codes, and quotes per theme and category

| **Theme** | | **Type** | **N** | **Description** | **Quote** |
| --- | --- | --- | --- | --- | --- |
| **Content** | | | | | |
| **Category: Barriers and facilitators** | | | | | |
|  | |  |  |  |  |
| Applicability of information | | R | 25 | Need for concrete applicable information | *‘’…how can you solve that?’’ (P11)* |
| Tone of voice | | Ne | 16 | Style in which the barriers and facilitators are written | *‘’It is very much written in policy language.’’ (P7)* |
| Credibility, barriers and facilitators | | P | 14 | Reliability of the information provided in the barriers and facilitators | *‘’A lot of information gives me the feeling that it is all well-founded, as it is scientifically based with definitions and references.’’ (P8)* |
| Interesting content | | N | 13 | Attractiveness of the included content | *‘’I think the content is strong.’’ (P5)*  *‘’I find the information interesting.’’ (P4)* |
| Terminology of recommendations | | Ne | 13 | Clarity of the terminology used in the recommendations | *‘’Adherence is quite a difficult word.’’ (P8)* |
| Understandability, barriers and facilitators | | P | 11 | Level of understandability of the barriers and facilitators | *‘’I think they are clearly worded. I have a feeling that I’m going to click on it. It’s very short and concise.’’ (P4)* |
| Title recommendations | | N | 10 | Clarity and completeness of the titles of the recommendations | *‘’The headings could be clearer.’’ (P6)* |
| Relevance, barriers and facilitators | | P | 9 | Relevance of the included content | *‘’I definitely find them useful.’’ (P9)* |
| Incomplete content | | Ne | 6 | Superficiality of information for users experienced with low-SEP groups | *‘’You just don’t know if it’s complete…the uncertainty of ‘have I seen it all?’’’ (P4)* |
| **Category: General** | |  |  |  |  |
|  | |  |  |  |  |
| Text general | | Ne | 21 | Amount of text on the website | *‘’I haven’t read it all; that takes me too much time.’’ (P2)* |
| Lengthy text | | R | 14 | Avoiding lengthy text to improve readability | *‘’I wouldn’t start with so much text.’’ (P10)* |
| Missing information | | N | 10 | Completeness of website content | *‘’Sometimes I feel like I am missing or unable to find information on the website. But maybe it does.’’ (P4)* |
| Importance of implementation | | R | 9 | Including information about implementation | *‘’It is part of its development, but it is also a huge success factor for the use of eHealth, and how you implement it is most certainly different for the low SEP.’’ (P5)* |
| Expansion of information | | R | 7 | Expanding the information in the website | *‘’However, I believe there is still room for more content.’’ (P5)* |
| Terminology general | | N | 6 | Terminology used on the website | *‘’I wonder if the word ‘end users’ is common in healthcare.’’ (P4)* |
| **Category: Stories** | | | | | |
|  |  | | | | |
| Realism | | R | 22 | Need for realistic stories | *‘’I would like to see real practical examples in case studies.’’ (P5)* |
| Empathy | | P | 16 | Enabling users to empathize with low-SEP groups | *‘’It is beautifully written; what I really like is that you make it very personal here, so that’s nice.’’ (P3)* |
| Applicability | | R | 16 | Inclusion of applicable knowledge in the stories | *‘’I think it could be very useful for humans. But the more concrete the examples are, in my opinion, the more useful they become. ’’ (P10)* |
| Expansion of stories | | R | 14 | Need for different variants of case studies | *‘’Yes, one persona is not enough.’’ (P4)* |
| Understandability | | P | 12 | Level of understandability of the stories | *‘’Yes, I understand them easily.’’ (P7)* |
| Examples | | P | 11 | Providing examples | *‘’I am curious about this fictional app.’’ (P11)* |
| Helpfulness | | N | 9 | Extent to which users of the website can benefit from the stories | *‘’So, I definitely find them useful. So I’d tell you probably yes, but think very carefully about what they should look like.’’ (P8)* |
| Abstract information | | R | 9 | Interest in abstract information | *‘’My mind works better with more abstract or conceptual information than with examples.’’ (P4)* |
| Conciseness | | R | 6 | Avoiding lengthy text to improve readability | *‘’I wouldn’t read it all the way through I think. I think I would like to know, in short, who is he?’’ (P6)* |
| Credibility | | Ne | 4 | Basing the stories on reliable information | *‘’I would also really like to know on the basis of what were the practical examples created. I can’t find that.’’ (P4)* |
| Stigmatization | | Ne | 2 | Extent to which users find the stories stigmatizing | *‘’Ronnie is of Surinamese descent. Well, that’s true of course. But it seems a bit stereotypical to me. I’m not sure if that’s the point.’’ (P8)* |
| Real examples | | R | 3 | Inclusion of real examples in the stories | *‘’I saw the Food Choice. I saw a sample? So the app, well, I don’t know if it already exists, or if it’s actually still a kind of prototype.’’ (P11)* |
| **System** | | | | | |
| **Category: Barriers and facilitators** | | | | | |
| Barriers and facilitators order | | Ne | 20 | Deterrent effect of the representation of the barriers | *‘’… and indeed what I just said, seeing a lot of barriers among each other is a deterrent, all those exclamation marks among each other.’’ (P6)* |
| Findability, barriers | | N | 14 | Findability of the barriers on the website | *‘’I have to conclude for myself that these are the barriers, and so I don’t think that is completely clear.” (P2)* |
| Findability, facilitators | | N | 13 | Findability of the facilitators on the website | *‘’That’s pretty easy; since I just need look at it from where I am now, then it’s just clicking.’’ (P2)* |
| Visual appeal | | Ne | 10 | Missing visuals and using unappealing icons | *‘’This sign also makes me feel as if something is really wrong.’’ (P7)* |
| Visual elements | | R | 10 | Making the barriers and facilitators pages more attractive | *‘’If you have different icons, this would be useful.’’ (P9)* |
| Order of facilitators | | R | 10 | Starting with facilitators instead of barriers | *‘’In my opinion, it would be better turned around. The positive side should be approached first.’’ (P4)* |
| Level of information | | R | 8 | Organizing content to make the information usable and understandable | *‘’There’s a possibility to get a bit bigger and fuller if it’s your last level.’’ (P9)* |
| **Category: General** | | | | | |
|  | |  |  |  |  |
| Onboarding | | R | 30 | Starting from the recommendations overview | *‘’I saw that overview at the beginning; I actually keep going back to it.’’ (P11)* |
| Usefulness of the scheme | | P | 14 | Navigating the website through the five-phase schedule | *‘’I really like the overview. So in a few steps where you should look at.’’ (P5)* |
| Visual appeal | | R | 14 | Making the website more attractive through images, colors, etc. | *‘’Yes, I also think it would really help to indicate it visually.’’ (P3)* |
| Website attractiveness | | N | 10 | Low website attractiveness | *‘’I think I expected a little more visually.’’ (P9)* |
| High-effort navigation | | N | 6 | Number of actions required to find the desired information | *‘’You really don’t have to go down that much. I don’t like scrolling that much.’’ (P10)* |
| Easy navigation | | P | 5 | Ease of website navigation | *‘’I notice that I know where to click much better now, and that’s gone very smoothly.’’ (P6)* |
| Easier navigation | | R | 5 | Easy navigation | *‘’A bit fewer clicks...’’ (P6)* |
| **Category: Practice stories** | | | | | |
|  |  | | | | |
| Unappealing presentation | | N | 7 | Unattractive layout | *“…you really have to read the small print below.” (P8)* |
| Perspective Low SEP important | | P | 5 | Perception of low-SEP groups | *“Yes, I think the personal story immediately grabs you and then you think, oh yes, we have to pay attention to that.” (P5)* |
| Visual elements | | P | 5 | Appeal of the layout page of the stories | *“That’s very nice to see. Nice, picture simple, rewards.’’ (P11)* |
| Videos | | R | 2 | Need to use videos | *“Practical examples…yes, I want to see a film there.” (P9)* |
| **Service** | | | | | |
| **Category: Credibility** | | | | | |
|  |  | | | | |
| Background information | | N | 13 | Referencing sources | *‘’I see references again. That’s nice.’’ (P8)* |
| Credibility issues | | Ne | 9 | Enhancing credibility of the website | *‘’Contact is the only thing that is not filled in. That surprised me a bit!’’ (P5)* |
| Proof of Credibility | | R | 7 | Managing expectations of the website | *‘’For a guideline, I would look at how can I make my apps low SEP-proof. Maybe I can also add a kind of checkmark; this is low SEP-proof.’’ (P3)* |
| **Category: General** | | | | | |
| Applicability of guide | | P | 40 | Applicability of the website in practice | *‘’It is good community knowledge to understand more about it.’’ (P5)* |
| User-profile tailoring | | R | 26 | Adapting the website to the users’ needs | *‘’Maybe you could do another one with layers so you have a basic version with clickable pieces and then an advanced user layer.’’ (P8)* |
| Credibility | | R | 21 | Demonstrating the credibility of the website | *‘’Yes, maybe it could be a little clearer who all this information comes from. Just you as researchers are connected to the university, things like that.’’ (P2)* |
| Recommendation of guide | | P | 14 | Recommending the website to others | *‘’So, yes, I would recommend it everywhere I can.’’ (P11)* |
| Community | | R | 11 | Incorporating existing user knowledge into the website | *‘’Imagine, I have a barrier, where else can I add it?’’ (P11)* |
| References | | R | 7 | Linking to existing websites | *‘’So a link to ‘Pharos’, maybe, or to other parties that are working on this, or good examples, front runners or initiatives or research groups.’’ (P5)* |
| **Category: Title guide** | |  |  |  |  |
| Guide | | R | 14 | Replacing guideline with guide | *‘’It is a guidance or a method description, isn’t it? Because it’s not about doing: do this, do that.’’ (P7)* |
| Guideline not fitting | | Neg | 10 | Calling the website a guideline | *‘’Hmm, I’m still a little confused about the word ‘guideline;’ that seems like a kind of checklist, and that is not your website.’’ (P7)* |
| Guide titles | | R | 5 | Name suggestions for the guide | *‘’Development tool.’’ (P6) ‘’Open eHealth.’’ (P5)* |

(N = Neutral, R = Future recommendation, Ne = Negative, P = Positive, F = frequency of code)
