# Supplementary material for: Guide Development for eHealth Interventions Targeting People With a Low Socioeconomic Position: Participatory Design Approach
Source: J Med Internet Res. 2023 Dec 4;25:e48461. doi: 10.2196/48461 (PMC10728791; doi:10.2196/48461)
Supplement: Multimedia Appendix 5 [file jmir_v25i1e48461_app5.docx]

# Multimedia Appendix 5 – Inclusive eHealth Guide final version and references to requirements

*The numbers 1, 2, etc. indicate the requirements that have been integrated into the final version.*

### Home page


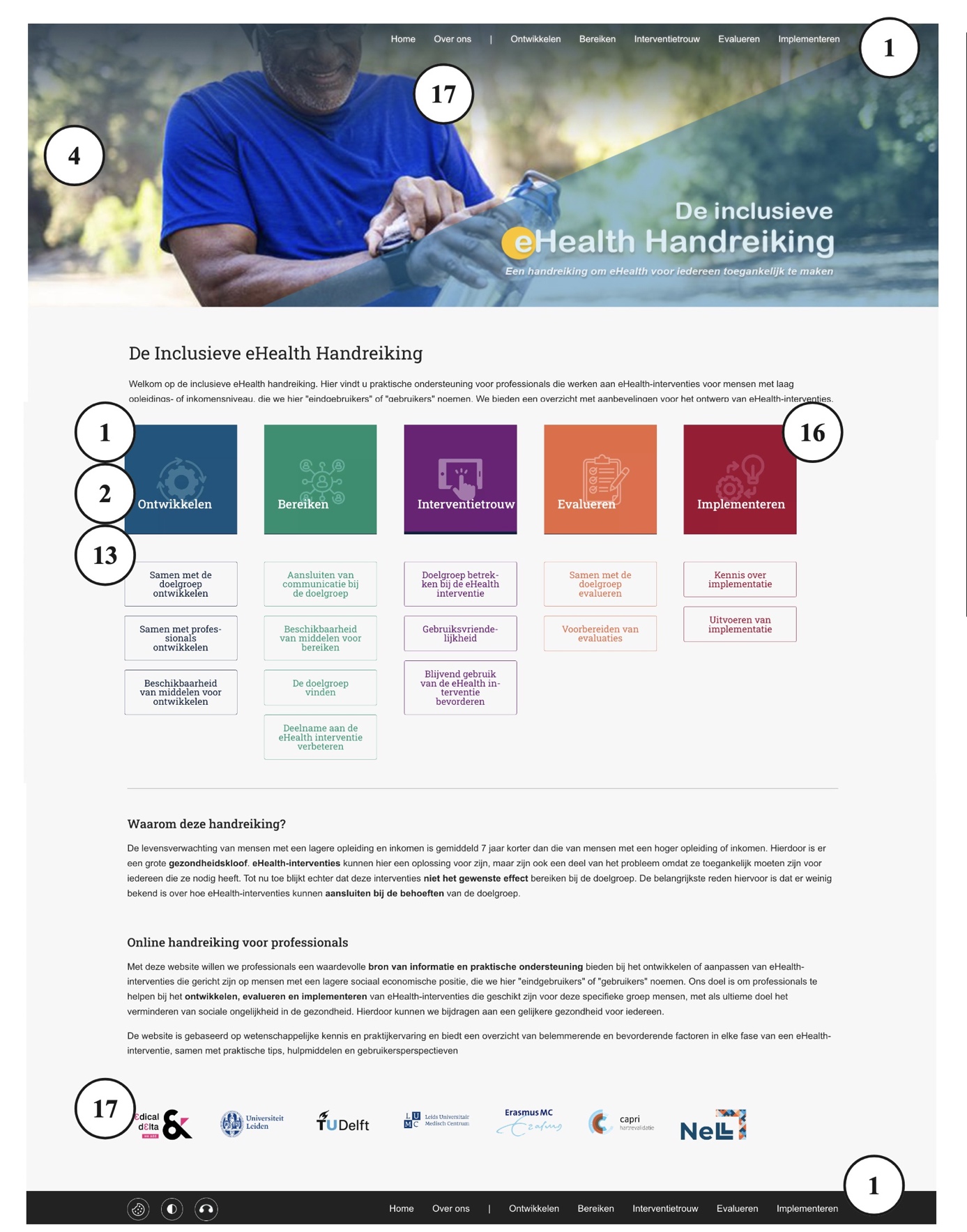


(1) Open Navigation - The website's navigation provides a starting point for users to explore the different phases of eHealth development. The navigation patterns are open, and users can find the phases at the top and bottom of the website, allowing them to explore other content directly.

(2) Starting scheme - Upon landing on the website, users are presented with a starting scheme that provides an overview of the content and helps them determine what content is most useful for them.

(4) Visual elements - A visual banner on the home page improves the appeal of the website and invites users to explore further.

(13) Suitability for different professionals - The comprehensive overview of the entire process of eHealth development makes the guide suitable for different types of professionals at various stages of eHealth development.

(16) Focus on implementation - The implementation section is dedicated entirely to addressing the needs of professionals looking for this type of information

(17) Enhance credibility –A dedicated 'About Us' page that includes information about the guide's background and the team behind it, as well as logos of the parties involved. This will help to improve the guide's credibility.

### Theme page

**
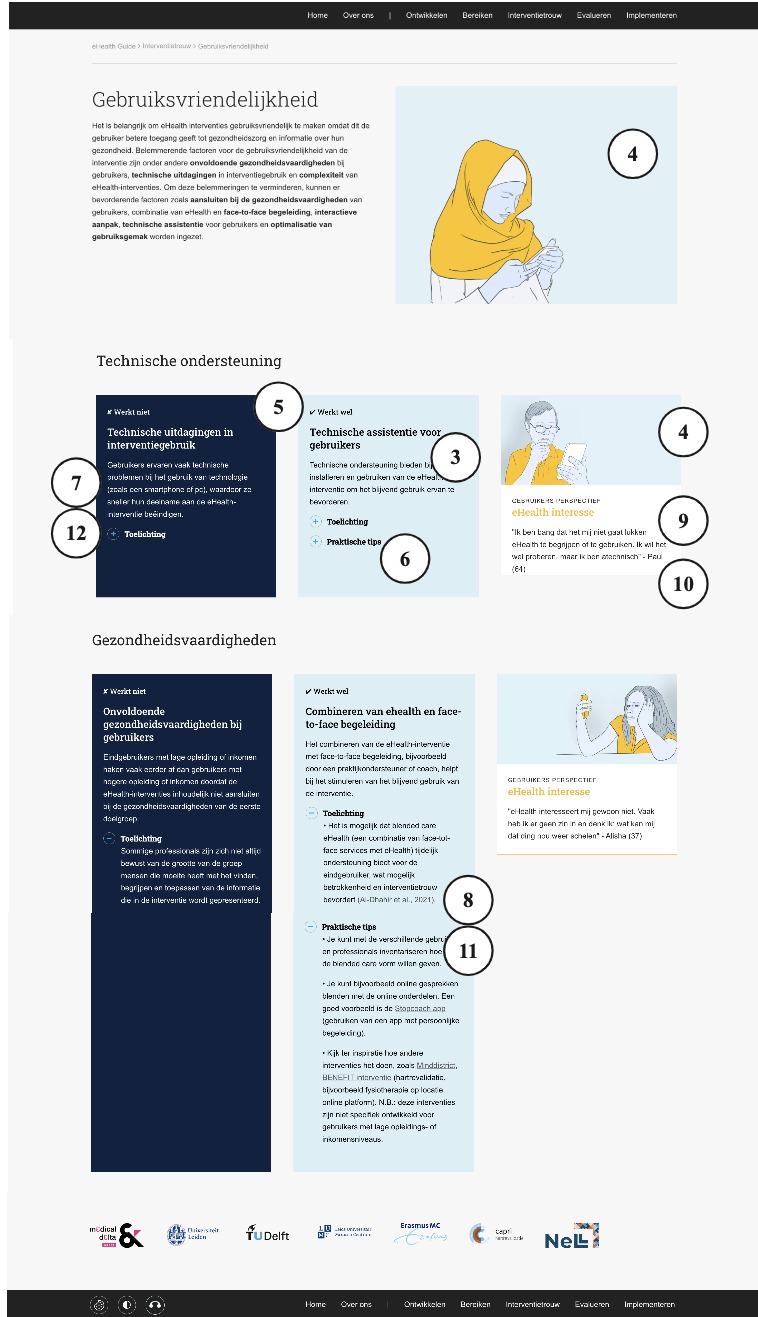
**

(3) Specific information - The starting scheme directs users to theme pages that offer specific information on barriers, facilitators, and user perspectives related to that theme.

(4) Visual elements - Illustrations of users in relevant contexts enhance the page's appeal.

(5) Concurrent presentation - Presenting both barriers and facilitators maintains a neutral tone and provides a comprehensive understanding of the information.

(6) Shorter pages - The use of "accordion" elements allows users to expand information selectively, keeping the page length shorter.

(7) Comprehensible information - The content is developed in collaboration with a communication expert to ensure it is easy to understand.

(8) Scientific evidence - The website cites scientific sources to back up information presented throughout.

(9) Realistic user representation - Using real quotes from previous user research provides a sense of realism.

(10) Abstract user information - User theme titles communicate the user's attitude in a clear way.

(11) Practical application - The website provides practical tips and references to external tools and resources to improve the practical applicability of the information.

(12) Informal tone-of-voice - An informal tone-of-voice is used throughout the guide, making it more approachable and engaging.
